# Impact and Effectiveness of the Quadrivalent Human Papillomavirus Vaccine: A Systematic Review of 10 Years of Real-world Experience

**DOI:** 10.1093/cid/ciw354

**Published:** 2016-05-26

**Authors:** Suzanne M. Garland, Susanne K. Kjaer, Nubia Muñoz, Stan L. Block, Darron R. Brown, Mark J. DiNubile, Brianna R. Lindsay, Barbara J. Kuter, Gonzalo Perez, Geraldine Dominiak-Felden, Alfred J. Saah, Rosybel Drury, Rituparna Das, Christine Velicer

**Affiliations:** 1 Royal Women's Hospital, University of Melbourne, Murdoch Childrens Research Institute, Victoria, Australia; 2 Danish Cancer Society Research Center and Department of Gynecology, Rigshospitalet, University of Copenhagen, Denmark; 3 Colombian National Institute of Cancer, Bogota; 4 Kentucky Pediatric and Adult Research, Bardstown; 5 Indiana University School of Medicine, Indianapolis; 6 Merck & Co, Inc, Kenilworth, New Jersey; 7 Universidad del Rosario, Bogota, Colombia; 8 Sanofi Pasteur MSD, Lyon, France

**Keywords:** HPV vaccination, cervical cancer, CIN, genital warts, Gardasil/Silgard

## Abstract

Prophylactic human papillomavirus (HPV) vaccination programs constitute major public health initiatives worldwide. We assessed the global effect of quadrivalent HPV (4vHPV) vaccination on HPV infection and disease. PubMed and Embase were systematically searched for peer-reviewed articles from January 2007 through February 2016 to identify observational studies reporting the impact or effectiveness of 4vHPV vaccination on infection, anogenital warts, and cervical cancer or precancerous lesions. Over the last decade, the impact of HPV vaccination in real-world settings has become increasingly evident, especially among girls vaccinated before HPV exposure in countries with high vaccine uptake. Maximal reductions of approximately 90% for HPV 6/11/16/18 infection, approximately 90% for genital warts, approximately 45% for low-grade cytological cervical abnormalities, and approximately 85% for high-grade histologically proven cervical abnormalities have been reported. The full public health potential of HPV vaccination is not yet realized. HPV-related disease remains a significant source of morbidity and mortality in developing and developed nations, underscoring the need for HPV vaccination programs with high population coverage.

## Abstract (precis)

This systematic review assessed the global impact and effectiveness of quadrivalent human papillomavirus (HPV) vaccination on HPV infection and disease in real-world settings over a decade of use. Substantial reductions in HPV 6/11/16/18 infection, anogenital warts, and cervical lesions have been achieved.

Human papillomavirus (HPV) is the commonest sexually transmitted virus worldwide, with first infection typically occurring soon after sexual debut. HPV-related diseases cause substantial morbidity and mortality globally [1]. Cervical cancer is the fourth most frequent cancer in women, with an estimated 530 000 new cases in 2012, accounting for 270 000 deaths (7.5% of all female cancer deaths) [2]. HPV infection causes virtually all cervical cancers and high-grade dysplasias, plus approximately 90% of anal, 70% of vaginal, 50% of penile, 40% of vulvar, and 13%–72% of oropharyngeal cancers [3–13]. The high-risk HPV genotypes 16 and 18 cause approximately 70% of cervical cancers and 80%–90% of HPV-related neoplasms at other sites, and the low-risk HPV genotypes 6 and 11 account for 90% of anogenital warts [3, 5, 8, 14, 15].

Prophylactic HPV vaccines in widespread use include the bivalent (2vHPV; Cervarix, GSK, Rixensart, Belgium) and quadrivalent (4vHPV; Gardasil/Silgard, Merck, Kenilworth, New Jersey) vaccines. A nonavalent (9vHPV; Gardasil 9, Merck) vaccine has recently been approved in several countries. All vaccines target HPV 16/18, whereas the 4vHPV vaccine also targets HPV 6/11 and the 9vHPV vaccine adds 5 oncogenic types (31/33/45/52/58) [14–17].

The 4vHPV vaccine was first licensed in Gabon in March 2006, then Mexico, Australia, and the United States in June 2006, followed by Europe in September 2006, and is now approved in 129 countries [18]. Although >60 countries include HPV vaccines in their national immunization programs, coverage rates vary (Supplementary Table 1). While specific indications differ somewhat by country, the 4vHPV vaccine is widely approved to prevent persistent infection with HPV 6/11/16/18, low- and high-grade cervical intraepithelial neoplasia (CIN1 and CIN2/3, respectively), adenocarcinoma in situ (AIS), cervical cancer, high-grade vaginal and vulvar intraepithelial neoplasia (VaIN2/3 and VIN2/3, respectively), vaginal and vulvar cancer, high-grade anal intraepithelial neoplasia (AIN2/3), anal cancer, and anogenital warts. Because vaccination is most protective when administered before HPV exposure, it is routinely recommended during preadolescence (usually age 11–12 years). Concurrent catch-up vaccination programs for older ages broaden coverage.

Programmatic implementation differs by country (Supplementary Table 1). An illustration of a pragmatic approach quickly translating into impressive reductions in HPV infections and related diseases due to vaccine types is the ongoing school-based vaccination program in Australia. This initiative, which began by temporarily offering free vaccine to females 12–26 years of age, was broadly endorsed and achieved high coverage of both school-aged girls and the older catch-up age group.

The 4vHPV vaccine was originally tested and approved as a 3-dose regimen, with a dosing schedule of 0, 2, and 6 months. More recently, a 2-dose schedule (6 or 12 months apart) has been recommended by the World Health Organization for younger age groups (eg, 9–14 years old at first dose), because immunogenicity with 2 doses in preadolescent and early adolescent girls was noninferior to antibody responses in women 16–26 years of age receiving 3 doses [16].

More than 205 million doses of 4vHPV vaccine had been distributed worldwide as of 31 December 2015. Although high efficacy against multiple endpoints was consistently observed in clinical trials [19], it is essential to document how trial results translate to real-world settings. Benefits have been described from various areas of the world subsequent to initiation of HPV vaccination programs [20]. Vaccine impact first became apparent for HPV infections and genital warts, which have short incubation periods following exposure to HPV [21]. Effects on cervical lesions, which take longer to develop, are now being documented, starting in Australia where cytological screening is instituted at a younger age than in most countries. Cancer rates are expected to decline only in the longer term, because carcinogenesis after HPV infection may require several decades to become manifest.

Both vaccine effectiveness and impact aim at evaluating “real-life benefit” and are typically measured through observational studies [22] (Supplementary Appendix II). Vaccine effectiveness corresponds to the proportion of infection or disease prevented among vaccinated individuals, and is estimated by comparing the incidence in vaccinated vs unvaccinated individuals within similar populations. Vaccine impact denotes the population-prevented fraction of infection or disease and is assessed by comparing prevalence or incidence in the vaccine era to a comparable population from the prevaccine era or by measuring population-level trends over time.

This descriptive study coincides with the 10th anniversary of the 4vHPV vaccine, which is the most widely used HPV vaccine in many countries around the world. Our systematic review comprehensively synthesizes available real-world data to quantify the reported effectiveness and impact of 4vHPV vaccination on HPV infection, anogenital warts, and cervical cytological and histological abnormalities.

## METHODS

PubMed and Embase databases were searched on 29 February 2016 for peer-reviewed manuscripts in any language published after 1 January 2007 using prespecified search terms (Supplementary Appendix I) [23, 24]. Observational studies of effectiveness or impact of 4vHPV vaccination on HPV infection or disease were considered for inclusion. We also searched the reference lists of retrieved articles for articles not identified initially. Studies reporting exclusively on the 2vHPV vaccine, review articles, data only in abstract form, burden-of-disease reports with no vaccine data, health economic modeling/simulation, and awareness studies were excluded. At least 2 reviewers examined articles to confirm inclusion criteria were satisfied and to reach consensus when necessary. The heterogeneity of study designs and the individual circumstances surrounding each study preclude summary estimates; consequently, our review is largely descriptive in nature.

## RESULTS

### Search Results

**Figure 1.**
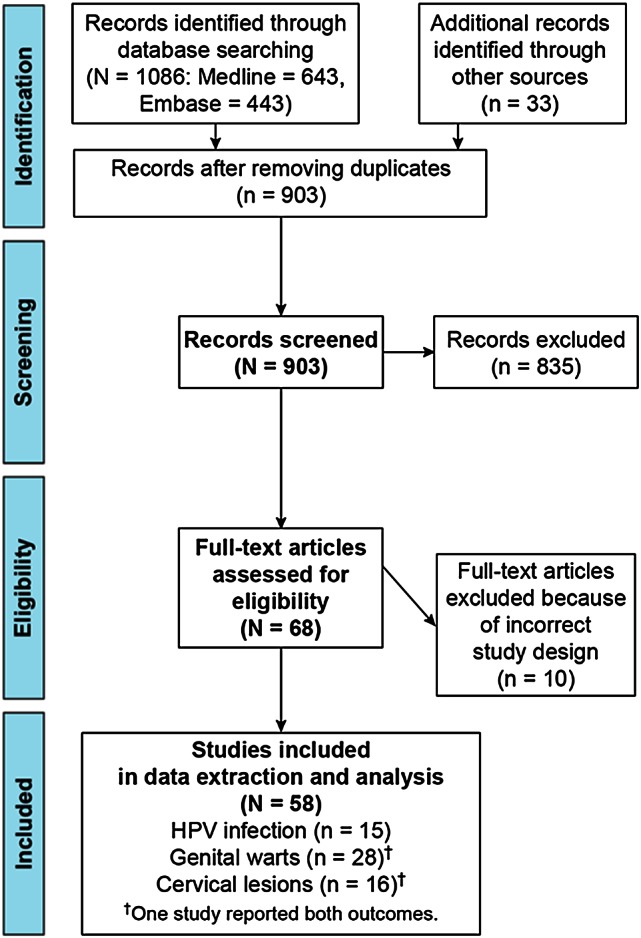
PRISMA (Preferred Reporting Items for Systematic Reviews and Meta-Analyses) diagram. Details of literature search and extraction for our systematic review. Abbreviation: HPV, human papillomavirus.

After screening 903 articles (Figure 1), 58 publications (6.4%) reporting the impact or effectiveness of 4vHPV vaccination on HPV infection, genital warts, and low- and high-grade cervical lesions from 9 countries satisfied the prespecified inclusion criteria (Table 1). Full citations for these 58 articles [A1–A58] are provided in Supplementary Appendix II, along with brief summaries and methodological critiques (Supplementary Table 2). Data pertaining to cervical cancer were not identified as vaccinated cohorts have not yet reached ages when cervical cancer is typically diagnosed.

**Table 1.** Summary of Publications Reporting the Impact and Effectiveness of Quadrivalent Human Papillomavirus Vaccination Programs in 9 Countries

Country (No. of Included Publications) and HPV Vaccination Program	Publications (No.) per Endpoint^a^			
	Genital Warts	HPV Infection	Cervical Cytological Abnormalities	Cervical Histological Abnormalities
**Australia** (18 publications)	**10**	**3**	**5**	**5**
Since April 2007: ongoing publicly funded school-based national program, girls aged 12–13 y Up to December 2009: school-based catch-up for females aged 12–17 y and community-based catch-up for women aged 18–26 y Since February 2013: ongoing school-based national program for boys aged 12–13 y, with catch-up 14–15 y in 2013–2014^b^	Fairley 2009 [A29] Donovan 2011 [A28] Read 2011 [A37] Ali 2013 [A23, A24] Liu 2014 [A34] Harrison 2014 [A31] Chow 2015 [A27] Smith 2015 [A39], 2016 [A43]	Tabrizi 2012 [A4], 2014 [A5] Chow 2015 [A8]	Brotherton 2011 [A53] Gertig 2013 [A46] Crowe 2014 [A45] Brotherton 2015 [A44, A54]	Brotherton 2011 [A53] Gertig 2013 [A46] Crowe 2014 [A45] Brotherton 2015 [A44, A54]
**Belgium** (2 publications)	**1**	**1**	…	…
November 2007: females 12–15 y reimbursed End of 2008: reimbursement extended to age 18 y Since 2010/2011: school-based, girls aged 12–13 y	Dominiak-Felden 2015 [A17]	Merckx 2014 [A14]		
**Canada** (3 publications)	**1**	…	**2**	**1**
Since 2007–2009: school-based, targeting girls grades 4–8 in all provinces/territories	Smith 2015 [A20]		Mahmud 2014 [A47] Smith 2015 [A20]	Ogilvie 2015 [A58]
**Denmark** (8 publications)	**5**	…	**2**	**3**
2006: licensed October 2008: 1st catch-up, females aged 13–15 y, free Since 2009: females aged 12 y, free August 2012: 2nd catch-up, females aged ≤27 y old	Baandrup 2013 [A25] Blomberg 2013 [A21] Sando 2014 [A38] Blomberg 2015 [A16] Bollerup 2016 [A42]		Baldur-Felskov 2014 [A48, A51]	Baldur-Felskov 2014 [A48, A51], 2015 [A52]
**France** (1 publication)	**1**	…	…	…
Initially: recommended for females ≥14 y old with no prior sexual intercourse or within 1st year following sexual debut Since September 2012: recommended in females aged 11–14 y, with catch-up for females 15–19 y	Judlin 2015 [A32]			
**Germany** (2 publications)	**1**	**1**	…	…
Since 2007: females aged 13–17, free	Mikolajczyk 2013 [A35]	Delere 2014 [A2]		
**New Zealand** (2 publications)	**2**	…	…	…
September 2008: vaccine available February 2009: school program for females aged 12–13 y, with catch-up until 2010 for females <20 y	Oliphant 2011 [A36] Wilson 2014 [A40]			
**Sweden** (5 publications)	**3**	**1**	…	**1**
2006–2011: public subsidy for on-demand vaccination, females aged 13–17 y Since 2012: organized, publicly funded school-based vaccination of females aged 10–12 y with catch-up for females 13–18 y	Leval 2012 [A33], 2013 [A19] Herweijer 2014 [A18]	Soderlund-Strand 2014 [A13]		Herweijer 2016 [A50]
**United States** (17 publications)	**4**	**9**	…	**4**
Since 2006: US Advisory Committee on Immunization Practices recommended routine vaccination for females aged ≥11 y	Bauer 2012 [A26] Swedish 2012 [A22] Flagg 2013 [A30] Nsouli-Maktabi 2013 [A41]	Cummings 2012 [A1] Kahn 2012 [A11] Powell 2012 [A6]^c^ Schlecht 2012 [A3] Markowitz 2013 [A12] Wilson 2014 [A15] Dickson 2015 [A9] Dunne 2015 [A10] Markowitz 2016 [A7]		Jamal 2013 [A56] Niccolai 2013 [A57] Hariri 2015 [A49, A55]

Abbreviations: HPV, human papillomavirus vaccine; y, years.

^a^ Publications may appear in >1 column if the study addresses >1 outcome. Please see Supplementary Table 2 in Supplementary Appendix II for detailed citations.

^b^ National HPV Vaccination Program Register. Preliminary estimates of HPV vaccination coverage for males—school-based program, first year of program delivery (2013). Available at: http://www.hpvregister.org.au/research/coverage-data/preliminary-estimates-male-hpv-coverage-2013. Accessed 27 April 2015.

^c^ Although included here in the original literature search, this citation did not actually provide data relevant to HPV infection.

### Overall Trends

Subsequent to introduction of the 4vHPV vaccine, consistent decreases in the prevalence of HPV 6/11/16/18 cervical/vaginal infections, genital warts, low- and high-grade cytological abnormalities, CIN2, CIN3, and AIS were observed among females in their teens and 20s (age groups targeted by national immunization programs). Decreases were highest in younger birth cohorts, reflecting a lower likelihood of prevalent HPV infection at time of vaccination. Irrespective of study design, declines were detected within 4 years after vaccine availability, even in settings with comparatively low vaccine coverage.

The effectiveness and impact of 4vHPV vaccination in reducing HPV-related infection and disease across studies depended on vaccine coverage in the study population, age of birth cohorts for whom vaccination was targeted in each country, implementation and duration of a catch-up program to increase coverage in older age groups within the indicated age range, time between program initiation and measurement of impact, and length of follow-up time covered by the study (Supplementary Table 3). Consequently, variability in reported findings more likely reflects operational properties inherent to each study, rather than fundamental differences in vaccine effectiveness among populations with otherwise similar baseline characteristics. Despite the many variables involving study design, the extent of vaccine uptake in the population under study, the quality of the research, and coexistent circumstances, the general findings were remarkably consistent across studies.

### Reductions in HPV 6/11/16/18 Infection Prevalence

**Figure 2.**
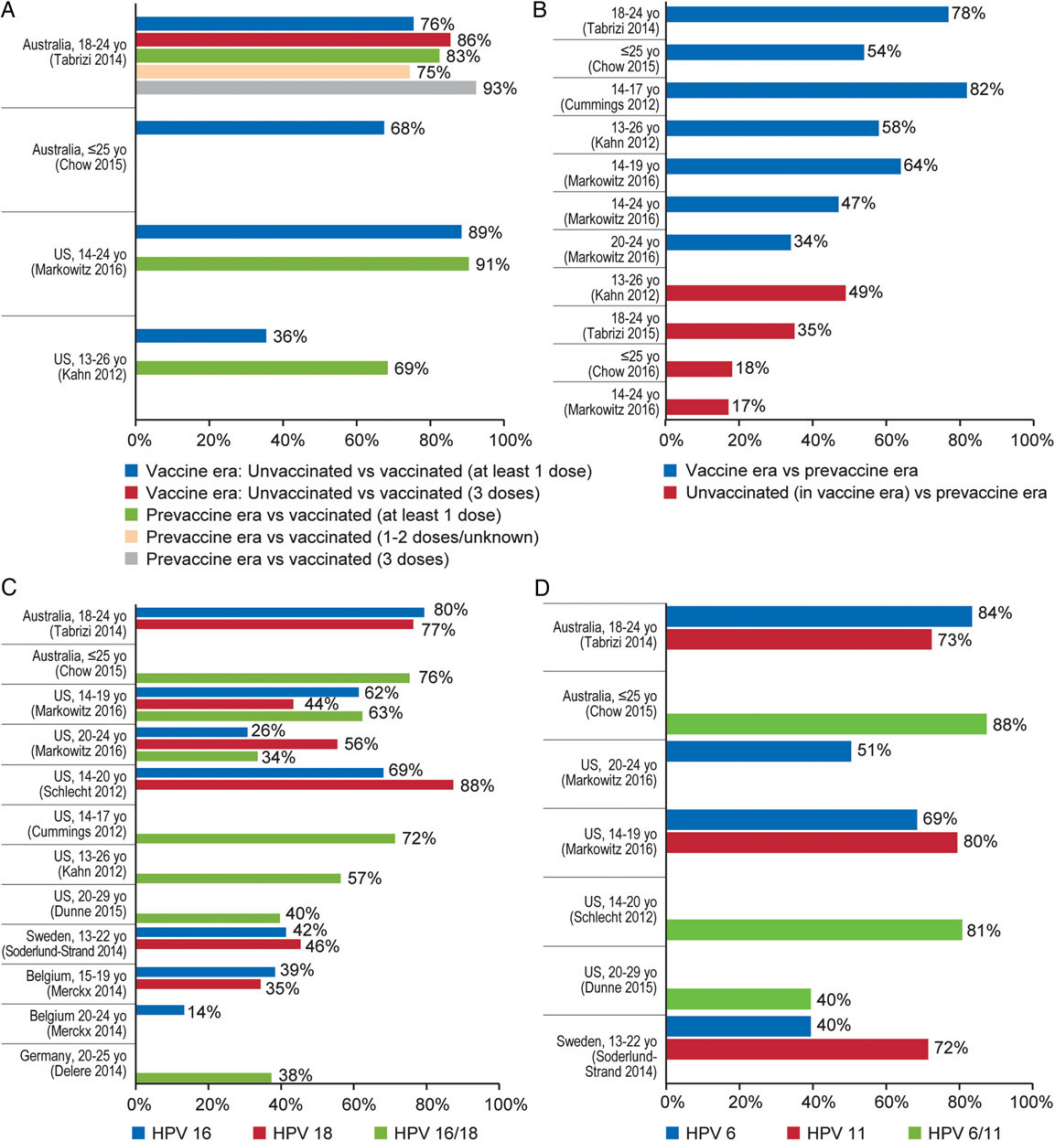
Impact and effectiveness of quadrivalent human papillomavirus (HPV) vaccination on prevalence of vaccine genotypes. *A*, Percentage reduction of prevalent HPV 6/11/16/18 infection among vaccinated females compared with prevaccine era or contemporaneous unvaccinated females. *B*, Percentage reduction of prevalent HPV 6/11/16/18 infection in vaccine era compared with prevaccine era. *C*, Percentage reduction of prevalent HPV 16/18 infection in vaccine era compared with prevaccine era or contemporaneous unvaccinated females. *D*, Percentage reduction of prevalent HPV 6/11 infection in vaccine era compared with prevaccine era. In Panel *A*, “2-doses” refers to an incomplete 3-dose schedule and not a primary 2-dose schedule. More details of the studies shown in the panels of Figure 2 are provided at the end of Supplementary Appendix II.

Within 6 years of 4vHPV vaccination availability, prevalent HPV 6/11/16/18 infections among Australian women 18–24 years of age decreased by 86% after 3 doses and by 76% after ≥1 dose, compared with contemporaneous unvaccinated women [A5, A8] (Figure 2*A*). In the United States, similar reductions (89%) within 6 years were reported in nationally representative samples of sexually active females aged 14–24 years who received ≥1 dose, in comparison to unvaccinated females in the vaccine era [A7]. In both countries, reductions were also reported when infection prevalence in vaccinated females was compared with the prevaccine era [A5, A7, A8] (Figure 2*B*). Additionally, Australia and the United States reported decreased infection prevalence among unvaccinated females in the vaccine era compared to the prevaccine era, evidence potentially reflecting herd protection (17%–49%).

Prevalent HPV 16/18 infections declined approximately 75%–80% among females aged ≤25 years in Australia in the vaccine era, in comparison to the prevaccine era [A5, A8], similar to several studies of US teenagers (62%–88%) [A1, A7] (Figure 2*C*). Among American women in their 20s, declines of 26%–56% were reported for prevalent HPV 16 and 18 infections [A7, A10], similar to declines (approximately 35%–45%) reported for females aged 13–22 years in Sweden [A13], 20–25 years in Germany [A2], and 15–19 years in Belgium [A14]. Smaller declines in prevalent HPV 16 and 18 infections (14%) were found among women 20–24 years of age in Belgium [A14].

Prevalent HPV 6/11 infections decreased approximately 75%–88% in Australian females ≤25 years old [A5, A8], 70%–80% in American teenagers [A3, A7], and 40%–50% in American women in their 20s in the vaccine era, in comparison to the prevaccine era [A7, A10]. Likewise, prevalent HPV 6 and 11 infection decreased by 40% and 72%, respectively, in Swedish females aged 13–22 years [A13] (Figure 2*D*).

### Genital Warts

Declines in the prevalence and incidence of genital warts tracked closely with decreases in HPV 6/11 infections [A17, A20, A23–A43] (Table 2). In countries with high vaccine uptake (such as Australia and Denmark), marked reductions in the frequency of genital warts were observed, particularly in the youngest age groups at vaccination (Supplementary Table 4). In women <21 years of age, yearly decreases of about 50% were observed in several studies [A25, A27, A37, A42], and up to 92.6% reduction was observed 4 years after vaccination program implementation in Australia [A24]. Furthermore, reductions were observed in unvaccinated young men, consistent with herd protection. In countries with moderate to low uptake of the vaccine at the time of the study (including France, United States, Canada, Sweden, Belgium, Germany, and New Zealand), the reduction in genital warts was lower (from 5.5% [A42] to 72.1% [A17]), varying widely depending on setting, age group, and time period considered. Reductions were mainly observed in young women targeted by vaccination programs, and evidence of herd protection was found in some studies [A17, A23, A24, A26, A36].

**Table 2.** Selected Examples of Percentage of Reduction in the Prevalence of Genital Warts in the Vaccine Era Compared to the Prevaccine Era or in Vaccinated Females Compared With Contemporaneous Unvaccinated Females

Country	Supplementary Reference	Setting	% Reduction in Genital Warts
Australia (high vaccine uptake)	Chow 2015 [A27]	Melbourne Sexual Health Centre, Victoria, within 7 y after start of vaccine era	45% annually among females <21 y
	Smith 2016 [A43]	Hospital admissions for genital warts from national database, within 4 y after start of vaccine era	85%–87%, 10–19 y 62%–67%, 20–29 y
	Donovan 2011 [A28]	National surveillance, within 2 y after start of vaccine era	59%, 12–26 y
Denmark	Bollerup 2016 [A42]	National prescription inpatient/outpatient registries, within 5 y after start of vaccine era	43% annually, 12–15 y 55% annually, 16–17 y 39% annually, 18–19 y 21% annually, 20–21 y 12% annually, 22–25 y 6% annually, 26–29 y
Sweden	Herjweijer 2016 [A18]	National hospital admissions that included genital warts diagnosis code, within 4 y after start of vaccine era	82%, 10–16 y (3 vs 0 dose) 71%, 10–16 y (2 vs 0 dose) 69%, 10–16 y (1 vs 0 dose)
United States	Flagg 2013 [A30]	Claims data (inpatient/outpatient visits or pharmacy dispensing) from large claims database (Truven Health Analytics), within 3 y after start of vaccine era	No change, 10–14 y 38%, 15–19 y 13%, 20–24 y

More details regarding the impact and effectiveness of quadrivalent human papillomavirus vaccination on anogenital warts are provided in Supplementary Tables 4 and 5, respectively, in Supplementary Appendix II.

Reductions in genital warts occurred as early as 1 year after program implementation in Australia [A29] and Germany [A35].

Abbreviation: y, years.

Generally high effectiveness against genital warts was observed for 3 vaccine doses (76%–93% [A16–A19]), with one exception [A20] where point estimates varied between 44% and 66% depending on specificity of the case definition (Supplementary Table 5).

### Cervical Cytological and Histological Abnormalities

**Figure 3.**
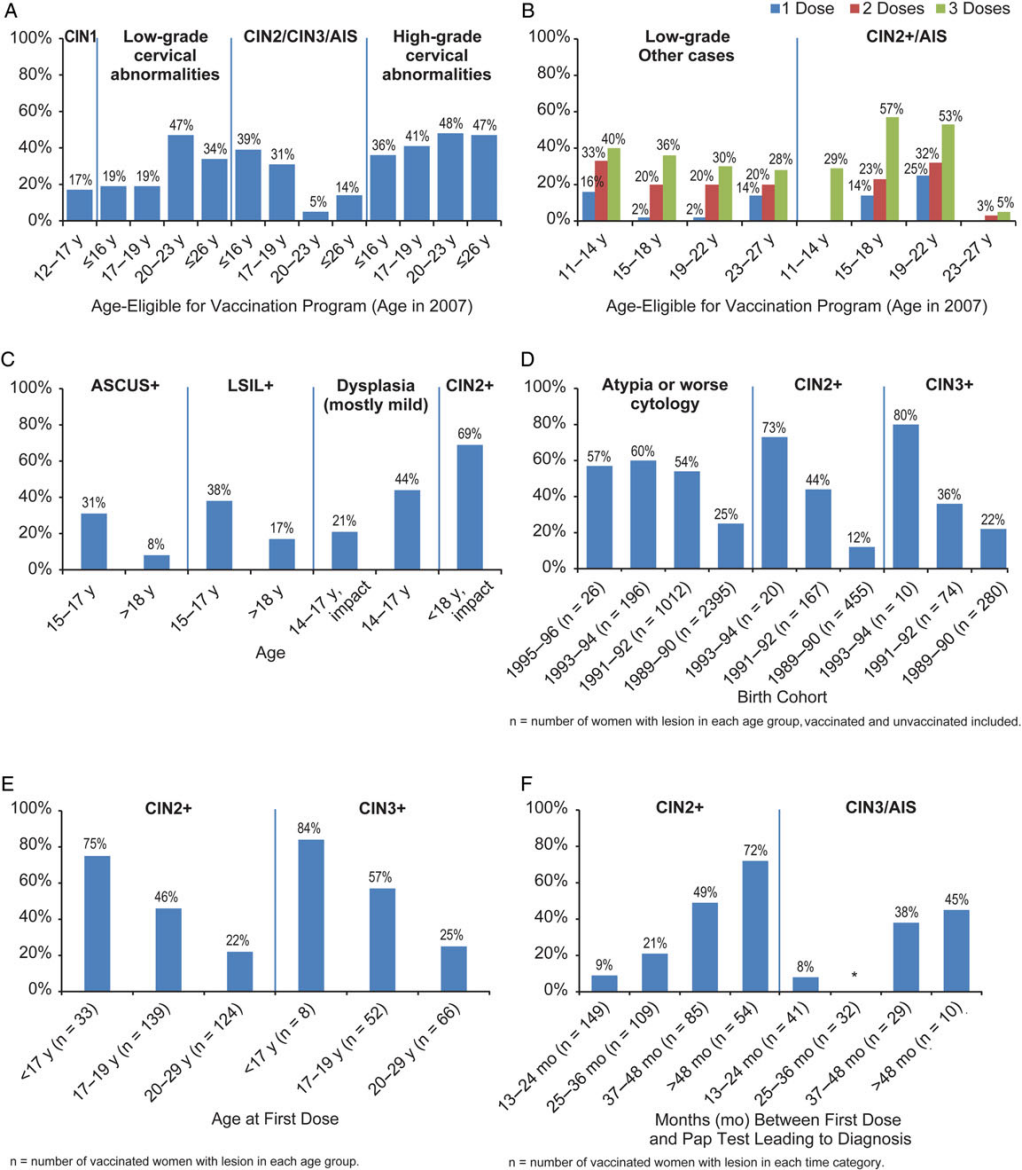
Impact and effectiveness of quadrivalent human papillomavirus (4vHPV) vaccination on cervical cytological and histological abnormalities. In Panel *B*, “2 doses” indicates receipt of an incomplete 3-dose schedule and not a primary 2-dose schedule. More details of the studies shown in the panels of Figure 3 are provided at the end of Supplementary Appendix II. *A*, Australia: population-based analysis of percentage reduction in cervical abnormalities among vaccinated (at least 1 dose) vs contemporaneous unvaccinated screened females in Victoria [A46, A44]. *B*, Australia: population-based analysis of percentage reduction in cervical abnormalities among vaccinated vs contemporaneous unvaccinated screened females in Queensland [A45]. *C*, Canada: percentage reduction in cervical abnormalities in vaccinated/vaccine era vs contemporaneous unvaccinated/prevaccine era in 3 provinces [A47, A20, A58]. *D*, Denmark: percentage reduction in cervical abnormalities in females vaccinated with 4vHPV vaccine (≥1 dose) vs unvaccinated women by birth cohort [A48]. *E*, Sweden: percentage reduction in CIN2+ and CIN3+ among females fully vaccinated with 4vHPV vaccine (3 doses) vs unvaccinated/partially vaccinated females, by age at first dose [A50]. *F*, United States: percentage reduction in HPV 16/18-related cervical abnormalities among females vaccinated with 4vHPV vaccine (at least 1 dose) vs contemporaneous unvaccinated females, by time between first dose and screening test leading to diagnosis; *null: adjusted prevalence ratio 1.02 [A49]. Abbreviations: AIS, adenocarcinoma in situ; ASCUS, atypical squamous cells of undetermined significance; CIN2/3, high-grade cervical intraepithelial neoplasia; LSIL, low-grade squamous intraepithelial lesion.

Within the first 5 years of Australia's 4vHPV vaccination program in Victoria, overall declines of 34% and 47% in low- and high-grade cervical cytological abnormalities, respectively, were evident in vaccinated cohorts of females 12–26 years of age at the start of the program, in comparison to unvaccinated females, with the largest declines in the 20- to 23-year-old age group (47% and 48%, respectively) [A44] (Figure 3*A*). Similar declines in low-grade abnormalities (20%–40%) among vaccinated (2–3 doses) compared with unvaccinated females aged 11–27 years in 2007 were also observed in Queensland, Australia [A45] (Figure 3*B*). Two studies of primarily low-grade cytological abnormalities in Canada [A20, A47] reported declines of approximately 20%–45% in vaccinated vs unvaccinated females aged 14–17 years (Figure 3*C*). Nationwide studies in Denmark reported 13%–33% estimated annual percentage declines in the incidence of cytological atypia or worse among 12- to 20-year-olds (impact) and 25%–60% declines in atypia or worse (effectiveness) [A48, A51] (Figure 3*D*). In each study, the greatest declines were among the younger age groups vaccinated with 2–3 doses. Only 1 study reported low-grade histological outcomes (CIN1) [A46], noting a 17% decrease among vaccinated vs unvaccinated Australian females 12–17 years of age in 2007 (Figure 3*A*).

For CIN2, CIN3, and AIS, the largest declines were found in younger cohorts and higher-grade lesions. In a nationwide Swedish analysis where vaccinated and unvaccinated women were followed for a mean of 2.6 and 5.1 years, respectively, declines of CIN2+ and CIN3+ among fully vaccinated females <17 years of age at vaccination were 75% and 84%, respectively, compared with unvaccinated and partially vaccinated females; in contrast, among those vaccinated between 20 and 29 years of age, declines in CIN2+ and CIN3+ were 22% and 25%, respectively [A50] (Figure 3*E*). Similarly, in a nationwide analysis from Denmark, respective declines for CIN2+ and CIN3+ of 73% and 80% were observed in the youngest birth cohort eligible for vaccination, and 12% and 22% in the oldest eligible birth cohort (born 1989–1990), compared with unvaccinated women [A48, A51] (Figure 3*D*). In Victoria, Australia, a similar age-related risk reduction was observed; among women vaccinated with ≥1 dose prior to their first cervical screening who were 12–26 years of age in 2007 with a mean age at vaccination of 21–22 years and an average follow-up <3 years, declines of CIN2/CIN3/AIS ranged from 39% to 5% in younger and older groups, respectively (Figure 3*A*), in comparison to unvaccinated women [A44]. In contrast, in Queensland, Australia, among females 15–18 years of age in 2007 vaccinated with 3 doses followed for approximately 2 years, a 57% reduction in CIN2+/AIS compared with unvaccinated women [A45] was reported, whereas a 5% reduction among females 23–27 years of age was reported in the same study (Figure 3*B*). In a US study of women with a mean age at vaccination of 22 years given ≥1 dose, the greatest declines for HPV 16/18-related CIN3/AIS (45%) and CIN2+ (72%) in comparison to unvaccinated women were found when the Pap test leading to the diagnosis occurred >4 years after vaccination [A49] (Figure 3*F*). A Canadian impact study found a 69% decline in CIN2+ in screened 15- to 17-year-olds after implementation of the HPV vaccination program (compared with the prevaccine era), but <6% reduction among females aged 18–22 years, who were ineligible for vaccination under the school-based program [A58] (Figure 3*C*).

## DISCUSSION

Prophylactic HPV vaccine programs constitute major public health initiatives worldwide. We assessed the global real-world impact and effectiveness of the 4vHPV vaccine over its first decade of use by systematically searching pertinent peer-reviewed literature. Estimates of 4vHPV vaccine effectiveness generally corresponded to vaccine efficacy results from clinical trials [20–22]. Rapid reductions up to approximately 90% in HPV 6/11/16/18 infections and genital warts after introduction of 4vHPV vaccination programs were first demonstrated in young women in Australia, Europe, North America, and New Zealand. Availability of population-based comparison data from the prevaccine era [25, A7] facilitated demonstration of impact. In Australia and the United States, decreases in the prevalence of HPV 6/11/16/18 infection and genital warts became evident <4 years after vaccine availability [A5, A12]. Subsequently, as successive birth cohorts began cervical screening, reductions as high as approximately 45% in low-grade cytological abnormalities and approximately 85% in high-grade histologically confirmed cervical lesions became apparent. For example, in Australia and Denmark where programs had achieved high and timely coverage with catch-up vaccination, respective reductions as high as 57% in CIN2+ and 80% in CIN3+ lesions were reported in the youngest cohorts vaccinated shortly after program implementation.

### Caveats and Limitations

Whereas 4vHPV vaccination has consistently been successful in reducing HPV 6/11/16/18 infection and disease, reported estimates of impact vary widely. In addition to the actual effectiveness of the vaccine itself, assessment of population impact depends on multiple factors including the breadth of the immunization program (cohort and catch-up), completeness and accuracy of data sources, availability and utilization of screening programs, the outcome under study, and vaccine coverage in females and males [26–30] [Supplementary Table 3]. Vaccine coverage may in turn be affected by national priorities and funding, access to healthcare, civil unrest, sexual practices, and vaccine acceptance. These elements together determine the measured impact in given populations. In this context, extrapolations across studies should be interpreted cautiously, with an emphasis on general trends rather than specific estimates of percentage of reduction.

In some regions, both the 4vHPV and 2vHPV vaccines were administered to a variable extent; however, the 4vHPV vaccine was predominantly used in all reports in the current review. Subsequent to 4vHPV vaccine introduction for females, some countries (including the United States, Australia, Austria, and Switzerland) have initiated HPV vaccination programs for males as a gender-neutral approach.

The anticipated benefit of vaccination on HPV-related cancer rates cannot be fully determined yet, because of the long latency periods following exposure. As vaccinated girls reach the age of cervical cancer screening, optimal cost-effective approaches to primary (preexposure vaccination) and secondary (periodic screening) prevention will need to be rethought [31, 32]. Some countries such as Australia are already planning to change the age, method, and frequency of screening in response to the high uptake and impact of HPV vaccines [33, 34] (Supplementary Appendix II).

### Future Challenges

The full potential of HPV vaccination is unfortunately far from being realized. Despite development of efficacious prophylactic vaccines, HPV-related diseases continue to present major public health challenges for both developing and developed nations. HPV vaccination rates appear to be lowest in low-income countries with the highest ongoing burden of HPV-related diseases. Globally, only 6.2% of females reaching 15 years of age in 2014 have received the vaccine [35], even with licensure in 129 countries, with 64 countries having HPV vaccines in their national immunization programs [18] (Supplementary Table 1). Worldwide, >600 000 new cancer cases are attributable to HPV annually, and HPV still causes nearly 10% of all new cancers in women worldwide, disproportionately affecting women in developing-world regions where 86% of incident cervical cancer cases and 88% of cancer deaths occur [36].

The World Health Organization recognizes the importance of cervical cancer and other HPV-related diseases as global public health threats and has reiterated its recommendation that HPV vaccines should be included in national immunization programs, provided that prevention of cervical cancer and/or other HPV-related diseases constitutes a public-health priority, vaccine introduction is programmatically feasible, sustainable financing can be secured, and the cost-effectiveness of vaccination strategies in the region is considered [37].

Many diverse hurdles remain before the global burden of HPV-related diseases can be further reduced. Unfounded notions about vaccine-related adverse experiences have derailed implementation of HPV vaccination programs in some countries, despite the positive safety profile observed over a decade of 4vHPV vaccine use and >200 million doses distributed [38, 39]. Although no intervention is without some risk, the challenge for public health officials and individual caregivers is to counterbalance the perceived vaccine-safety concerns against the unacceptably high numbers of women and men currently suffering and dying from HPV-related cancers. Explicit physician recommendations and healthcare provider reminder/recall systems could serve to improve vaccination rates. Integrating HPV vaccination with new, more sensitive cervical screening assays as part of routine primary preventive care will improve healthcare for all women.

## CONCLUSIONS

Over the last decade, the impact of the 4vHPV vaccine in real-world settings has become increasingly documented and is attributable to high vaccine effectiveness in targeted populations with high coverage [20, 21]. The greatest impact has been seen where the vaccine is routinely administered before HPV exposure. Vaccination of catch-up cohorts has accelerated observed benefits. Disappointingly, preventable HPV-related diseases persist, underscoring the need for wide-reaching HPV vaccination programs with high population coverage prior to HPV exposure. Universal adoption of safe and effective prophylactic HPV vaccine programs targeting girls and boys before sexual debut can prevent the substantial morbidity and mortality still attributable to HPV worldwide [20, 21, 32, 40]. Ensuring broad coverage of appropriate populations can provide a major advancement in global public health.

## Supplementary Data

Supplementary materials are available at http://cid.oxfordjournals.org. Consisting of data provided by the author to benefit the reader, the posted materials are not copyedited and are the sole responsibility of the author, so questions or comments should be addressed to the author.

Supplementary Data
