# Supplementary material for: Impact and Effectiveness of the Quadrivalent Human Papillomavirus Vaccine: A Systematic Review of 10 Years of Real-world Experience
Source: Clin Infect Dis. 2016 May 26;63(4):519–27. doi: 10.1093/cid/ciw354 (PMC4967609; doi:10.1093/cid/ciw354)
Supplement: Supplementary Data [file supp_63_4_519__index.html]

Impact and Effectiveness of the Quadrivalent Human Papillomavirus Vaccine: A Systematic Review of 10 Years of Real-world Experience — Supplementary Data 

# Impact and Effectiveness of the Quadrivalent Human Papillomavirus Vaccine: A Systematic Review of 10 Years of Real-world Experience

## Supplementary Data

Supplementary Data

- Supplementary data\_appendix1 - docx file
- Supplementary data\_appendix2 - docx file
