# Supplementary material for: Impact and Effectiveness of the Quadrivalent Human Papillomavirus Vaccine: A Systematic Review of 10 Years of Real-world Experience
Source: Clin Infect Dis. 2016 May 26;63(4):519–27. doi: 10.1093/cid/ciw354 (PMC4967609; doi:10.1093/cid/ciw354)
Supplement: Supplementary Data [file supp_ciw354_ciw354supp_data_appendix1.docx]

**SUPPLEMENTAL APPENDIX I:**

**Protocol for Literature Search and Extraction**

**Impact and Effectiveness**

**Of the Quadrivalent Human Papillomavirus Vaccine:**

**A Systematic Review of Ten Years of Real-World Experience**

**Suzanne M. Garland^1^, Susanne K. Kjaer^2^, Nubia Muñoz^3^, Stan L. Block^4^,**

**Darron R. Brown^5^, Mark J. DiNubile^6^, Brianna R. Lindsay^6^, Barbara J. Kuter^6^, Gonzalo Perez^6,7^, Geraldine Dominiak-Felden^8^, Alfred J. Saah^6^,**

**Rosybel Drury^8^, Rituparna Das^6^, and Christine Velicer^6^**

**^1^The Royal Women's Hospital, University of Melbourne, Murdoch Childrens Research Institute, Melbourne, Victoria, Australia**

**^2^Danish Cancer Society Research Center and Department of Gynecology, Rigshospitalet, University of Copenhagen, Copenhagen, Denmark**

**^3^Colombian National Institute of Cancer, Bogota, Colombia**

**^4^Kentucky Pediatric and Adult Research, Bardstown, KY, USA**

**^5^ Indiana University School of Medicine, Indianapolis, IN, USA**

**^6^Merck & Co., Inc., Kenilworth, NJ, USA**

**^7^Universidad del Rosario, Bogota, Colombia**

**^8^Sanofi Pasteur MSD, Lyon, France**

**LITERATURE SEARCH and EXTRACTION PROTOCOL**

With the assistance of P95 (a healthcare research company specializing in pharmaco-epidemiology), we searched PubMed and Embase on 19-November-2015 for peer-reviewed manuscripts in any language published after 1-January-2007 using prespecified search terms (see protocol below). As our review was nearing completion, the databases were re-searched using the same procedures to capture the most recent relevant publications through 29-February-2016.

**Background**

Because of the tenth anniversary of the approval of Gardasil in 2016, SPMSD wants to perform a systematic literature review of the real- life benefit (*i.e*., impact/effectiveness) of Gardasil. SPMSD has asked P95 to assist with this review specifically in the data collection aspect (literature searching). This protocol therefore refers exclusively to the data collection (literature searching) and not the data analysis.

**Objectives**

The objective of this project is to review published study reports on the real-life benefit (i.e. impact/effectiveness) of Gardasil in countries/regions where Gardasil vaccination has been implemented.

**Methods**

***Types of studies: inclusion/exclusion criteria***

We will include published peer-reviewed studies reporting impact and/or effectiveness of Gardasil in cervical lesions, genital warts, and HPV infections.

For the purpose of this research, "impact" will be defined as an evaluation of the population impact of *HPV vaccination program* in a country. It refers to a potential change in the disease burden of the endpoint of interest following the introduction of a vaccination program. It is usually evaluated in an ecologic manner (before vs after evaluation/analysis of trends over time) and is to be interpreted with the vaccine coverage level reached within the study population. Therefore, burden of disease studies with no concurrent vaccine uptake (also sometime qualified as "impact"), health-economic impact studies, models, simulations, extrapolations, awareness, and/or acceptability studies will be excluded.

For the purpose of this research, "effectiveness" will be defined as an evaluation of the direct protective effect of the vaccine in subjects vaccinated in a real-life setting (in comparison to unvaccinated subjects). It will thus refer to *observational* studies only. The results of long-term follow-up studies of clinical trial (which results may also be presented as "effectiveness") will be excluded.

Studies retrieved mentioning Cervarix will not be excluded during the search, but will be excluded during selection if no data on Gardasil alone are presented. Review papers will be excluded; however, original studies discussed in the review will be included.

***Electronic searches***

The search strategies below will be used to search Medline (via PubMed) and EMBASE. There will be no language restrictions.

**Medline (PubMed)**

A) Intervention

1. ("human papillomavirus vaccine L1, type 6,11,16,18"[Supplementary Concept] OR "human papillomavirus vaccine L1, type 6,11,16,18"[All Fields] OR "gardasil"[All Fields])

2. "quadrivalent hpv vaccine"[all fields] OR “qHPV”[all fields] OR “HPV4”[all fields] OR “4HPV”[all fields] OR “4VHPV”[all fields]

3. 1 OR 2 = ("quadrivalent hpv vaccine"[all fields] OR “qHPV”[all fields] OR “HPV4”[all fields] OR “4HPV”[all fields] OR “4VHPV”[all fields] OR "human papillomavirus vaccine L1, type 6,11,16,18"[Supplementary Concept] OR "human papillomavirus vaccine L1, type 6,11,16,18"[All Fields] OR "gardasil"[All Fields] OR "silgard"[All Fields])

B) Study types

4. Impact OR effectiveness OR epidemiological monitoring [MeSH] OR population surveillance [Mesh] OR program evaluation [MeSH] OR real-life OR routine-use OR incidence OR prevalence

C) Final search strategy

5. Final intervention string AND Final study type string

("quadrivalent hpv vaccine"[all fields] OR “qHPV”[all fields] OR “HPV4”[all fields] OR “4HPV”[all fields] OR “4VHPV”[all fields] OR "human papillomavirus vaccine L1, type 6,11,16,18"[Supplementary Concept] OR "human papillomavirus vaccine L1, type 6,11,16,18"[All Fields] OR "gardasil"[All Fields] OR "silgard"[All Fields]) AND (Impact OR effectiveness OR epidemiological monitoring [MeSH] OR population surveillance [Mesh] OR program evaluation [MeSH] OR real-life OR routine-use OR incidence OR prevalence)

**EMBASE**

A) Intervention

(‘quadrivalent hpv vaccine’ OR Gardasil OR HPV4 OR silgard/tn)

B) Study types

(‘epidemiological monitoring’/exp OR ‘population surveillance’/exp OR ‘program evaluation’/exp OR Impact OR Effectiveness OR Real-life OR Routine-use OR Incidence OR Prevalence)

C) Final search strategy

(‘quadrivalent hpv vaccine’ OR Gardasil OR HPV4 OR silgard/tn) AND (‘epidemiological monitoring’/exp OR ‘population surveillance’/exp OR ‘program evaluation’/exp OR Impact OR Effectiveness OR Real-life OR Routine-use OR Incidence OR Prevalence)

**Alternative search strategies**

To the list of studies retrieved from the electronic searches we will add further studies in two ways:

1. Retrospective analysis – hand search of the references in the bibliography of all (appropriate) papers
2. Prospective analysis – hand search of all papers referring to the (appropriate) paper, to be found using Google Scholar

**Data collection and extraction**

***Selection of studies***

Two P95 employees (MB, SJ) will independently review the list of studies obtained from the electronic searches to identify studies that fulfill the selection criteria. These papers will be listed in an output table, providing the following fields: authors, title, source, abstract and/or PubMed reference code (PMID number), endpoint, method, and country. The output table agreed upon is attached.

PRISMA diagram (stage 1)

As the output table is built, the flow of publications will be noted. Based on these numbers, a preliminary PRISMA diagram will be prepared.

***Data extraction and management***

P95 will conduct the data extraction and management (including critical appraisal of studies). Exclusion of studies at this stage will be noted with reason for exclusion.

PRISMA diagram (stage 2)

To finalize the PRISMA diagram, Sanofi Pasteur MSD will communicate the flow of publications during the data extraction phase to P95, so these numbers can be included.

**Postscript**

To consolidate the relevant literature in a consistent format, results are reported as the percent reduction in each outcome (HPV infections, genital warts, cytological and histological abnormalities) for each publication. Some estimates rely on small sample sizes and may be unstable or imprecise. Accordingly, trends in results (by age, dose number, etc.) should be interpreted as a general indication of trends. When publications reported more than one type of estimate, we used the following hierarchy: (i) adjusted vaccine effectiveness; (ii) unadjusted vaccine effectiveness; (iii) adjusted relative risks/hazard ratios/odds ratios/prevalence ratios; (iv) relative risks/hazard ratios/odds ratios/prevalence ratios; (v) adjusted/weighted prevalence data; and (vi) raw prevalence data.
