# Supplementary material for: Impact and Effectiveness of the Quadrivalent Human Papillomavirus Vaccine: A Systematic Review of 10 Years of Real-world Experience
Source: Clin Infect Dis. 2016 May 26;63(4):519–27. doi: 10.1093/cid/ciw354 (PMC4967609; doi:10.1093/cid/ciw354)
Supplement: Supplementary Data [file supp_ciw354_ciw354supp_data_appendix2.docx]

**SUPPLEMENTAL APPENDIX II:**

**Additional Description, Tables, and Figures**

**Impact and Effectiveness**

**Of the Quadrivalent Human Papillomavirus Vaccine:**

**A Systematic Review of Ten Years of Real-World Experience**

**Suzanne M. Garland^1^, Susanne K. Kjaer^2^, Nubia Muñoz^3^, Stan L. Block^4^,**

**Darron R. Brown^5^, Mark J. DiNubile^6^, Brianna R. Lindsay^6^, Barbara J. Kuter^6^, Gonzalo Perez^6,7^, Geraldine Dominiak-Felden^8^, Alfred J. Saah^6^,**

**Rosybel Drury^8^, Rituparna Das^6^, and Christine Velicer^6^**

**^1^The Royal Women's Hospital, University of Melbourne, Murdoch Childrens Research Institute, Melbourne, Victoria, Australia**

**^2^Danish Cancer Society Research Center and Department of Gynecology, Rigshospitalet, University of Copenhagen, Copenhagen, Denmark**

**^3^Colombian National Institute of Cancer, Bogota, Colombia**

**^4^Kentucky Pediatric and Adult Research, Bardstown, KY, USA**

**^5^ Indiana University School of Medicine, Indianapolis, IN, USA**

**^6^Merck & Co., Inc., Kenilworth, NJ, USA**

**^7^Universidad del Rosario, Bogota, Colombia**

**^8^Sanofi Pasteur MSD, Lyon, France**

**Impact versus Effectiveness**

The important but often blurred distinction between effectiveness (on an individual level) and impact (on an ecologic or population level) is worth explicitly drawing with respect to study endpoints. An impact study evaluates the population impact of an HPV vaccination program in a country or demarcated locale, quantifying the potential change in the disease burden reflecting the endpoints of interest prior and subsequent to introduction/implementation of a vaccination program. Impact is usually evaluated in an ecologic manner (before vs after comparison/analysis of trends over time) and should be interpreted in the context of the duration of the program and the level of vaccine coverage achieved in the particular study population. In contrast, an effectiveness study represents an observational (non-interventional) evaluation of the direct protective effect of the vaccine in subjects vaccinated in a real-life setting compared to unvaccinated subjects. Impact is usually determined using cross-sectional data, whereas effectiveness can be operationally assessed using retrospective or prospective longitudinal data if individual vaccination status can be ascertained.

Assessment of population impact depends on multiple covariates in addition to the effectiveness of the vaccine itself, including the breadth of the immunization program (cohort and catch-up), aspects of the targeted population, vaccine coverage in females and males, age at vaccination, population dynamics, the extent of herd protection, the interval between the start of the program and the time of measurement, and the specific outcome variable being assessed as well as the availability and utilization of screening programs. Vaccine coverage may in turn be affected by national priorities and funding, access to healthcare, civil unrest, sexual practices, and the usual age of initiation of sexual activity. All these elements together determine the overall impact on a given population. Furthermore, because many countries do not maintain national registries, ascertainment bias may confound the interpretation of the results.

Although quadrivalent HPV (4vHPV) vaccine programs have consistently been successful in reducing HPV 6/11/16/18 infection and HPV-related disease, reported estimates of impact and effectiveness vary regionally based on age at vaccination; inclusion of boys/men; vaccination coverage; the number and timing of vaccine doses; efforts at catch-up vaccination; secular trends; the timing and length of follow-up; the particular endpoint(s) under study; cervical cancer screening recommendations and practice; study design, population, size, and statistical power; types of surveillance programs (such as specific HPV-vaccine surveillance programs); completeness and accuracy of data sources and analytical methods, especially as to whether the baseline referent is the pre-vaccine era [26-30] (**Table S3**).

From a methodological standpoint, both impact and effectiveness studies have inherent strengths and limitations. Impact studies are ecological. The observed effect might thus be confounded by inadequately controlled elements that have changed between the pre- and post-vaccination period (such as data sources/reporting bias, case definition/ascertainment method, natural history of the disease, behavior norms, media awareness, and other public-health programs or policy).

Ecological fallacy occurs when an outcome is erroneously attributed to a particular exposure without consideration of other factors that may impact the measured association. For example, a study may report that the reduction observed in cervical lesions between the prevaccine and vaccine eras is attributable to vaccination when changes in screening practices concurrently occurring in the study population were not taken into account in the analyses. Consequently, the measured impact cannot always be confidently attributed to the vaccination program (unless vaccine coverage is high, the measured effect can be objectively and unambiguously identified, and no other important elements differ between the pre- and post-vaccination era). In this regard, reassuring evidence can be provided by an "external" comparator (*e.g.,* another sexually transmitted disease for HPV studies or the same endpoint in a similar population not targeted by the vaccination program). Of course, all factors potentially having an impact on the number of cases besides the vaccination program need to be conscientiously documented.

In contrast to impact studies, effectiveness studies are less subject to extraneous factors and generally considered to provide a higher level of evidence than impact studies. Effectiveness studies can result in a more robust measure of the effect of vaccination itself (including number of doses) on the health outcome of interest (*e.g.,* cervical lesions) as long as: selection and other biases do not influence the population receiving or not receiving the vaccine; case status is ascertained in the same manner irrespective of vaccination status (*e.g.,* the vaccinated and unvaccinated populations are similar in screening behavior and any follow-up associated with abnormal screening results); the data regarding vaccination, case status, and potential confounding variables are consistently measured and the associated datasets are complete.

**Herd protection in heterosexual/bisexual men from vaccination programs targeting women: emerging evidence**

                Reductions in HPV infection during the vaccine era could result from the direct and indirect effects of a vaccination program, or alternatively represent coincidental secular trends.  The Australian vaccination program initially targeting females could afford protection to male sexual partners of vaccinated women.  In a retrospective observational study of young heterosexual men attending the Melbourne Sexual Health Centre for *Chlamydia trachomatis* infection, the dramatic decline in the HPV prevalence confined to vaccine genotypes among mostly unvaccinated, sexually active males over time implies that herd protection from widespread female vaccination is likely responsible [Chow PF, Machalek DA, Tabrizi SN, et al. Quadrivalent vaccine targeted human papillomavirus genotypes in heterosexual men after the Australian female human papillomavirus vaccination programme: a retrospective observational study. *Lancet Infect Dis* 2016; online June 6. <http://dx.doi.org/10.1016/S1473-3099(16)30116-5>].

**Changes in screening programs for cervical cancer: the Australian experience**

Pursuant to the dramatic reduction in HPV infection as well as in high-grade dysplasia in Australia, a review of the National Cervical Screening Program through what is known as the RENEWAL Program was undertaken [Australian Department of Health. National Cervical Screening Program.

Available at: http://hpv.health.gov.au/the-program/. Accessed 26 March 2016].  In the context of the vaccine era, the committee reexamined conventional cytological screening (which starts at 18 years of age or 2 years after sexual debut whichever is later and continues until 69 years of age, performed at 2-year intervals per NHMRC Guidelines).  This standard approach was compared to liquid-based cytology (LBC) with reflex HPV DNA testing or primary HPV DNA testing for women from age 25-64 years every 3-5 years. The RENEWAL program will involve a delay in the age of screening until 25 years and screening with an HPV DNA test with limited genotyping and this will be every five years until 74 years of age for those found to be negative.  Those with a positive HPV test will be followed with reflex LBC triage for HPV-vaccinated and unvaccinated alike and in accordance with the cervical screening pathway.  Those who are negative at 70-74 years of age will exit the screening program.  Women who are more than 74 years of age who have never been screened will be offered screening.  HPV and cytology co-testing is not recommended. Women who are under screened or never screened and who refuse participation or are unable to participate in the program will be encouraged to provide a self-collected sample for HPV analysis.

Commencing in May 2017, Australia will be using HPV DNA as a primary test with partial genotyping. Women positive for HPV 16/18 will go straight to colposcopy. If positive for other HPV types reflex LBC will be performed next; the next step and subsequent follow-up will depend on the cytological findings as informed by an algorithm still being refined. Economic analyses need to be performed to quantify actual reductions in direct and indirect healthcare costs. Incorporation of HPV vaccination into routine preventive care, along with advances in cancer screening, should positively impact healthcare for women while lowering costs.

**Screening program for juvenile onset recurrent respiratory papillomatosis (JORRP) in Australia**

JORRP is a rare but highly morbid respiratory condition caused by HPV 6 and 11. Surveillance for JORRP is actively underway in Australia.  Given the catch-up program and 10 years into the vaccine era, the rate of JORRP in the population is being followed through a surveillance system involving the Australian Paediatrics Surveillance Unit as well as ENT surgeons in the country. Baseline data has recently become available [30].

**Table S1. Countries Including HPV Vaccine in their National Immunization Programs (NIPs): Year Introduced, Target Age Groups, Delivery Method, and Coverage, 2006–2015^a^**

| **Region/**  **Country** | **Year**  **Introduced into NIP^1^** | **Target Age Group (Years) or**  **Grade ^b 1^** | **Catch-up Age Group (Years)^2^** | **Delivery**  **for**  **Primary**  **Target Group^2^** | **Estimated  3-dose**  **Coverage ^c^**  **(year evaluated)^2^** | **Comments** | |
| --- | --- | --- | --- | --- | --- | --- | --- |
| **EUROPE** |  |  |  |  |  |  | |
| Austria ^4,5^ | 2014 | 9-14 |  | Schools/PC | 62% for boys & girls (2015) | Recommendation given in 2007; funding & 2-dose schedule started in 2014 | |
| Belgium **^d, 6-8^** | 2007 | 12-13 | 13–18 | Varies by region | Varies by region: 30%-83%  (2012-2013) | Lower coverage in Flanders region, higher coverage in Wallonian region | |
| Bulgaria | 2012 | 12 |  |  |  |  | |
| Czech Republic **^13^** | 2012 | 13 |  | PC | 65% (Unknown) | Reimbursed – no official NIP | |
| Denmark **^14^** | 2009 | 12 | 13–15 | PC/Health centers | 82% (2015) | In August 2012 catch-up expanded to women up to 27 years | |
| Finland **^15^** | 2013 | 11-12 |  | Schools | 68% (2015) |  | |
| France **^16^** | 2007 | 11-14 | 15–23 | PC/Health centers | 17% for 16 year olds (2014) |  | |
| Germany **^17^** | 2007 | 9-14 |  | PC/Health centers | 16- 56% (2012) | Coverage by age: 14 yo - 16.3%; 15 yo - 37.7%;  16 yo - 45.9%; 17 yo - 55.6%.  Initial recommendation was for a vaccination age of 12 to 17 and 3-dose vaccination; STIKO recommendation since 2014 has been 2-dose vaccination for girls between the ages of 9 – 14 years. | |
| Greece | 2008 | 11-18 |  | PC/Health centers | Varies by source: 5%-27% (2011) |  | |
| Greenland | 2008 | 12 | 13–15 | Mixed |  |  | |
| Hungary ^18, 19^ | 2014 | 12 |  | Schools | 80% (2015) for 2-dose schedule |  | |
| Iceland | 2011 | 12 |  | Schools | 88% (2014) |  | |
| Ireland ^20^ | 2010 | 12–13 |  | PC/Health centers | 85% for 12-13 yo, 45% for  18-19 yo (2014) |  | |
| Italy ^22^ | 2007–2008 | 12 | Varies by region | PC/Health centers | 11-71%% (2014) | Coverage by age: 11 yo - 10.7%; 12 yo - 62.4%;  13 yo - 67.0%; 14 yo -71.1%; 15 yo - 72.1%; 16 yo - 70.9%; 17 yo - 70.8% | |
| Latvia | 2010 | 12 |  | Mixed | 61% (2011) |  | |
| Liechtenstein ^23^ |  | 11-14 | 15-19 |  |  |  | |
| Luxembourg ^8,24^ | 2008 | 12-18 |  | PC/Health centers | 29% (2008) |  | |
| Republic of Macedonia (formerly the Yugoslav Republic of Macedonia) | 2010 | 12 | 13–26 | Schools | 65% (2012) |  | |
| Netherlands | 2010 | 12 | 13–16 | Mixed | 61% (2014) |  | |
| Norway | 2009 | 12 |  | Schools | 79% (2014) |  | |
| Portugal | 2009 | 13 | 17 | PC/health centers | 87% (2015) |  | |
| Romania | 2010 | 12 |  | Mixed | <5% |  | |
| San Marino | 2009 | 11 |  |  |  |  | |
| Slovenia | 2009 | 12 |  | Schools | 49% (2012) |  | |
| Spain | 2008 | 11–14 |  | Varies by region | 73% (2014) |  | |
| Sweden | 2012 | 10–12 | 13-18 | Schools | 80% (2014) |  | |
| Switzerland | 2008 | 11-14 | 15-19 | Mixed | 51% (2013) |  | |
| United Kingdom | 2008 | 12–13 | 13–17 | Schools | 86% (2014) |  | |
|  |  |  |  |  |  |  | |
| **CENTRAL ASIA** |  |  |  |  |  |  | |
| Uzbekistan (GAVI) | 2015 |  |  |  |  |  | |
|  |  |  |  |  |  |  | |
| **AMERICAS** |  |  |  |  |  |  | |
| Argentina | 2011 | 11 |  | Mixed | 50% (2013) |  | |
| Canada ^e 11^ | 2007–2009 | 9-14 for females in 4 provinces & 3 territories; 9-14 for females & males in 5 provinces | Varies by province | Schools | 60 to 85% by region (2013) |  | |
| Barbados | 2014 | 11 |  |  |  |  | |
| Bermuda | 2008 | 11-13 |  |  |  |  | |
| Brazil | 2014 | 9 |  | Mixed |  | Also recommended in HIV+ population | |
| Cayman Islands |  | 11-13 |  |  |  |  | |
| Chile | 2015 | 9 | 11-12 | Schools |  | Also recommended in HIV+ population | |
| Colombia ^12^ | 2012 | 9-17 |  | Mixed | 87% (2013) |  | |
| Ecuador | 2014 | 9 |  | Clinics |  |  | |
| Guyana | 2011 | 11 |  |  |  |  | |
| Mexico ^e 25^ | 2008 | 10 |  | Mixed | 67% | Boys vaccinated in Mexico City | |
| Panama | 2008 | 10 |  | Mixed | 67% (2010) |  | |
| Paraguay | 2013 | 10 |  | Mixed |  |  | |
| Puerto Rico | 2006 | 11-18  females & males |  |  |  |  | |
| Peru | 2011 | 10 |  | Schools |  |  | |
| Surinam | 2013 | 9 |  |  |  |  | |
| Trinidad & Tobago | 2013 | 11-12 |  |  |  |  | |
| United States ^g 27^ | 2006 | 11–12 females & males (starting in 2011) | 13–26 | PC/Health centers | 40% for females, 22% for males, (2014) |  | |
| Uruguay | 2013 | 12 |  | Clinics (permissive) |  |  | |
|  |  |  |  |  |  |  | |
| **ASIA-PACIFIC** |  |  |  |  |  |  | |
| Australia^3^ | 2007 | 12-13 females (& males starting in 2013) | Up to 26 to end of 2009 (females)  14-15 year old catchup for boys (2013-14) | Schools | 73.1% girls (2014)  Slightly lower for boys |  | |
| Bhutan ^9^ | 2010 | 12 | 13–18 | Mixed | >90% (2014) |  | |
| Brunei | 2012-2015 | 12-13 |  |  |  |  | |
| Malaysia | 2010 | 13 | 13–18 | Schools | 87% (2011) |  | |
| Japan | 2011 | 13 |  | Health Centers | 0.6%  (2014 - Sapporo) |  | |
| Philippines | 2015 | 9 |  | Health Centers |  |  | |
|  |  |  |  |  |  |  | |
| **WESTERN PACIFIC** |  |  |  |  |  |  |  |
| Fiji ^h^ | 2008 | 13 |  |  |  |  |  |
| Kiribati | 2011 | NA |  |  |  |  |  |
| Federated States of Micronesia ^i^ | 2009 | 9 |  | PC/Health centers |  |  |  |
| Marshall Islands ^i^ | 2008 | 11-12 |  | PC/Health centers |  |  |  |
| New Zealand | 2008 | 12 | 13–18 | Mixed ^j^ | 56% (2014) |  |  |
| Palau ^i^ | 2009 | 9-26 |  | PC/Health centers |  |  |  |
| Singapore | 2010 | 9–26 |  | PC/Health centers |  |  |  |
|  |  |  |  |  |  |  |  |
| **EASTERN MEDITERRANEAN** |  |  |  |  |  |  |  |
| Abu Dhabi, United Arab Emirates | 2008 | 15–17 | 18–26 | Schools | 59% (2011) |  |  |
| Israel ^21^ | 2011 (females) & 2015 (males) | 14 (females) & 14(males) |  | Schools/Health centers | ~60% (2014) |  |  |
|  |  |  |  |  |  |  |  |
| **AFRICA** |  |  |  |  |  |  |  |
| Botswana ^10^ | 2015 | 9-13 |  | Schools/Health centers |  | 2-dose program; 3 doses for HIV positives |  |
| Lesotho | 2012 | 9-13 |  |  |  |  |  |
| Libya | 2013 | 15 |  |  |  |  |  |
| Rwanda (GAVI) | 2011 | PG 6 | In year 2 & 3, PG 6 &  SG 3 (9th school year) | Schools ^k^ | 99% (2013) |  |  |
| South Africa ^26^ | 2014 | 9 (PG4) |  | Schools | 87%  (Dose 1) |  |  |
| Republic of Seychelles | 2014 | 10-12 |  | Schools |  |  |  |
| Uganda (GAVI) | 2012 | 10 |  | Schools |  |  |  |

**FOOTNOTES**

PG: Primary school grade; SG: Secondary school grade; PC: Primary care providers; NA: Not available; Mixed: Schools plus primary care and health centers; yo: year old.
^a^ **Table S1** was in part adapted from Markowitz L *et al*. [Vaccine 305 (2012): F139-F148] and updated primarily from Bruni L *et al*. [Summary Report 2015-12-23]. Additional information for this table was obtained from other published data, presentations or personal communications as listed, reflecting the most current sources we could locate. Completeness and quality of the data may vary for the different countries and data may not always be fully up to date. Blank cells imply that data were unavailable at this time, not applicable, or unknown. Any oversights or inaccuracies in the data are unintentional.

^b^ Target age group in years. When a school grade is targeted, the age group desired for targeting is in parenthesis.

^c^ Data obtained from published data, official websites, or personal communication. Data were not available for all countries; different methods were used to evaluate vaccine coverage; comparisons between countries are not possible.

^d^ Healthcare in Belgium is the responsibility of three different communities: Flemish, Germanophonic, and French. Programs and policies vary by community. Coverage provided in table is for multiple communities.

^e^ Extended dosing schedule being used in Mexico and two provinces in Canada (Quebec and British Columbia): doses at 0, 6, and 60 months.

^f^ Two-dose coverage for the extended dosing schedule adopted in 2009, targeting girls aged 9–12 years for the first two doses.

^g^ In December 2011, quadrivalent HPV vaccine for males 11–12 years was included in the routine immunization schedule. Catch-up recommended through age 21 for those not previously vaccinated. In December 2015, 9-valent HPV vaccine for both females and males was included in the routine immunization schedule.

^h^ Unsuccessful initial introduction; reintroduced in 2012.

^i^ Personal communication, M. Larzelere, Project Officer, Immunization Services Division, CDC.

^j^ Mainly schools.

^k^ Out-of-school 12 year old girls were also targeted.

**Sources for Table S1**

For the year introduced, target age group, catchup age group, delivery method, and estimated coverage, references 1 and 2 were used unless more current data were provided in the references below (as noted in the table).

1. Markowitz L, Tsu V, Deeks SL et al. Human Papillomavirus Vaccine Introduction – The First Five Years. *Vaccine* 305 (2012): F139-F148.
2. Bruni L, Barrionuevo-Rosas L, Albero G et al. ICO Information Centre on HPV and Cancer (HPV Information Centre). Human papillomavirus and related diseases in the world. Summary Report 2015-12-23.
3. Australia: National HPV Vaccination Program Register. Preliminary estimates of HPV vaccination coverage for males - school based program, first year of program delivery (2013) 2015 [27 April 2015]. Available from: <http://www.hpvregister.org.au/research/coverage-data/preliminary-estimates-male-hpv-coverage-2013>
4. Austria: <http://www.bmg.gv.at/home/Impfplan>
5. Austria: Communication with Austrian Ministry of Health, E. Joura, Feb 11, 2016.
6. Belgium (Wallonie): Vermeeren A, Miermans MC, Swennen B. Evolution de 2008 à 2013 des couvertures vaccinales des enfants en âge scolaire en Fédération Wallonie-Bruxelles, Provac, Bruxelles 2014.
7. Belgium (Flanders): [www.infectieziektebulletin.be](http://www.infectieziektebulletin.be)
8. Belgium & Luxembourg: Arbyn M, Simoens C, Van Damme P et al. Introduction of human papillomavirus vaccination in Belgium, Luxembourg and the Netherlands. Gynecol Obstet Invest 2010; 70:224–232.
9. Bhutan: Dorji T, et al. Introduction of a National HPV vaccination program into Bhutan. *Vaccine* (2015), <http://dx.doi.org/10.1016/j.vaccine.2015.05.078>
10. Botswana: Personal communication with Ministry of Health, J. Millogo, January 14, 2016. anapress.com
11. Canada:

- <http://healthycanadians.gc.ca/healthy-living-vie-saine/immunization-immunisation/children-enfants/schedule-calendrier-table-1-eng.php>
- <http://www.gov.mb.ca/health/publichealth/cdc/docs/hpv_phn.pdf>
- [http://www.chrisd.ca/2015/10/21/human-papillomavirus-hpv-vaccine-boys-manitoba/#.VikIiP7ruUk](http://www.chrisd.ca/2015/10/21/human-papillomavirus-hpv-vaccine-boys-manitoba/)
- <http://www.msss.gouv.qc.ca/sujets/santepub/vaccination/index.php?aid=193>
- <http://publications.msss.gouv.qc.ca/acrobat/f/documentation/piq/html/web/Piq.htm> (10.4.4)
- <http://www.health.gov.on.ca/en/ms/hpv/immunize.aspx>
- <http://www.ehealthsask.ca/services/manuals/Documents/sim-chapter5.pdf>
- <http://novascotia.ca/dhw/populationhealth/documents/School-Based-Immunization-Coverage-Nova-Scotia.pdf>
- <http://www.hss.gov.nt.ca/sites/default/files/nwt_immunization_schedule_0.pdf>
- <http://www.phac-aspc.gc.ca/publicat/ccdr-rmtc/12vol38/acs-dcc-1/index-eng.php>
- <http://www.hss.gov.yk.ca/pdf/im-manual.section1.pdf>
- <http://www.bccdc.ca/health-info/immunization-vaccines/vaccines-in-bc/human-papillomavirus-(hpv)-vaccine>
- <http://www.fil-information.gouv.qc.ca/Pages/Article.aspx?aiguillage=ajd&type=1&idArticle=2312038377>
- Personal communications - PEI Department of Health and Wellness (Dec 1, 2015) (Website Update In-Progress)

1. Colombia: <https://www.minsalud.gov.co/Documentos%20y%20Publicaciones/Justificaci%C3%B3n%20de%20Vacunaci%C3%B3n%20contra%20el%20Virus%20del%20Papiloma%20Humano%20(VPH).pdf>
2. Czech Republic: <http://www.zakonprolidi.cz>
3. Denmark: SSI: <http://www.ssi.dk/Aktuelt/Temaer/Generelle%20temaer/Vaccination%20mod%20livmoderhalskraft.aspx>
4. Finland: Personal communication, T. Vesikari, Jan 2016.
5. France: InVS à partir de l’échantillon généraliste des bénéficiaires (EGB) <http://www.invs.sante.fr/Dossiers-thematiques/Maladies-infectieuses/Maladies-a-prevention-vaccinale/Couverture-vaccinale/Donnees/Papillomavirus-humains>
6. Germany: KIGGS Study.
7. Hungary: Epinfo - Epidemiológiai Információs Hetilap, March 2015 (Hungarian immunization schedule).
8. Hungary: <https://www.antsz.hu/phv/lakossagnak/orszagos_tisztifoorvos_takekoztato_hpv_vedooltas.html>
9. Ireland: Health Service Executive (HSE)
10. Israel: Personal communication, D. Greenberg, Jan 26, 2016.
11. Italy: Istituto Superiore di Sanita <http://www.epicentro.iss.it/problem/hpv/CopertureVaccinail.asp>
12. Liechtenstein: <http://vaccine-schedule.ecdc.europa.eu/Pages/Scheduler.aspx>
13. Luxembourg: <http://plancancer.lu/about/prevention/vaccination-hpv/>
14. Mexico: Programa de Vacunacion universal y semans Nacionales de salud. Lineamientos Generales 2016. Documento de la Secretaria de Slud, sub-secretaria de Prevencion y Promocion de la Salud. Centro Nacional para la Salud de la Infancia y la Adolescencia. <http://www.censia.salud.gob.mx/mwg-internal/de5fs23hu73ds/progress?id=QU7fadR6WV>
15. South Africa: WHO; Richter; [www.phassa.org.za](http://www.phassa.org.za)
16. United States: Reagan-Steiner S, Yankey D, Yeyarajah J et al. National, regional, state, and selected local area vaccination coverage among adolescents aged 13–17 Years — United States, 2014; *Morb Mortal Wkly Rep* 2015:64 (29): 784-792.**Table S2. Listing of Literature Citations Identified From the Systematic Search and Included in the Formal Analysis.**

| Reference #  Full Citation  Country | Data Source  Study Population  Sample Size | Data Collection Dates  Vaccination Program Comments | Strengths and Limitations |
| --- | --- | --- | --- |
| Effectiveness Against HPV Infection |  |  |  |
| A1  Cummings T, Zimet GD, Brown D, et al. Reduction of HPV infections through vaccination among at-risk urban adolescents. Vaccine 2012; 30: 5496-5499.  USA^1^ | Nested longitudinal study (Young Women's Project assessing risk and protective factors for STIs)  Females aged 14-17 in Indianapolis.  Historical cohort n=150, prospective cohort n=75 | Pre-vaccination: "Fall" 1999 to July 2005.  Post-vaccination: 2010-2010 (months not reported)  Vaccination program date not reported | Strengths: Apparent clinician-verified vaccination status; logistic regression incorporating paired random effects reported.  Limitations: Small sample size; 27% of females vaccinated with ≥2doses reported sexual activity prior to vaccination; small sample of high-risk females; clinician or self-sampled sample collection-possible bias. |
| A2  Delere Y, Remschmidt C, Leuschner J, et al. Human Papillomavirus prevalence and probable first effects of vaccination in 20 to 25 year old women in Germany: a population-based cross-sectional study via home-based self-sampling. BMC Infect Dis 2014; 14: 87.  Germany | Cross-sectional survey  Females aged 20-25, nationwide  Age 20-25 (n=735), 20-21 (n=213), 22-23 (n=288), 24-25 (n=234) | October 2010 to September 2012  All females aged 12-17 years have been eligible for vaccination since 2007. Both 4vHPV and 2vHPV vaccine are available in Germany. | Strengths: Population-based.  Limitations: Only 31% reported no sexual activity prior to vaccination; 12% response rate- suggesting possible self-selection bias; self-reported vaccination status; self-sampled specimens. |
| A3  Schlecht NF, Burk RD, Nucci-Sack A, et al. Cervical, anal and oral HPV in an adolescent inner-city health clinic providing free vaccinations. PLoS One 2012; 7: e37419.  USA^1^ | Cross-sectional survey  Females aged 12 to 19 attending Mount Sinai Adolescent Health Center in New York  n=645 sexually active inner-city young females | Not reported. Time can be inferred from date of publication (2012) and the text, "When compared to the general MSAHC clinic population (data not shown), the study cohort is younger (mean patient age in 2010 was 18.6 vs. 17.7 in this study)"  Vaccination program date not reported. | Strengths: Clinician-collected specimen; vaccination defined as 3 doses; results derived from general estimating equation with multivariate logistic regression.  Limitations: Calendar years not specified; 90% reported sexual intercourse prior to 1^st^ dose; high-risk population; unclear if vaccination self-report or from medical record. |
| Effectiveness Against HPV Infection |  |  |  |
| A4  Tabrizi SN, Brotherton JM, Kaldor JM, Skinner SR, Cummins E, Liu B, Bateson D, McNamee K, Garefalakis M, Garland SM. Fall in human papillomavirus prevalence following a national vaccination program. J Infect Dis 2012; 206: 1645-1651.  Australia^2^ | Repeat cross-sectional survey  Females nationwide aged 18-24 attending family planning clinics (34 sentinel clinical sites) in three major cities.  Pre-vaccine period n=202; post vaccine period mixed: vaccination status, n=404, vaccinated n=338, unvaccinated n=57 | Pre-vaccination period 2005-2007, post-vaccination period 2010-2011 | Refer to A5 for updated information. |
| A5  Tabrizi SN, Brotherton JM, Kaldor JM, et al. Assessment of herd immunity and cross-protection after a human papillomavirus vaccination programme in Australia: a repeat cross-sectional study. Lancet Infect Dis 2014; 14: 958-966.  Australia^2^ | Repeat cross-sectional survey  Females nationwide aged 18-24 attending family planning clinics (34 sentinel clinical sites) in three cities.  Pre-vaccine period n=202; post vaccine period mixed: vaccination status, n=1058, unvaccinated n=149, partly-vaccinated n=391, vaccinated n=518 | Pre-vaccine period n=202; post vaccine period mixed: vaccination status, n=1058, unvaccinated n=149, partly-vaccinated n=391, vaccinated n=518 | Strengths: Representative sample of females receiving Pap screening, self-reported vaccination status verified against National Vaccine Program Register, clinician-collected specimens, prevalence ratios and vaccine effectiveness adjusted for potential confounding variables.  Limitations: 48% vaccinated reported sexual activity prior to completion of the full vaccine series. |

| Reference #  Full Citation  Country | Data Source  Study Population  Sample Size | Data Collection Dates  Vaccination Program Comments | Strengths and Limitations |
| --- | --- | --- | --- |
| Effectiveness Against HPV Infection |  |  |  |
| A6  Powell SE, Hariri S, Steinau M, et al. Impact of human papillomavirus (HPV) vaccination on HPV 16/18-related prevalence in precancerous cervical lesions. Vaccine 2012; 31: 109-113.  USA^1^ | Indirect cohort study design. Data collected from the HPV-IMPACT project  Females 18–31 years diagnosed with CIN2+ reported from pathology laboratories in California, Connecticut, New York, Oregon and Tennessee.  5083 CIN2+ cases reported: 3855 had vaccination history investigated and 1900 had vaccine history documented. | 2008-2011 | Effectiveness study of lesions; does not report reduction rates for HPV infections. Refer to A49 for updated data. |

| Reference #  Full Citation  Country | Data Source  Study Population  Sample Size | Data Collection Dates  Vaccination Program Comments | Strengths and Limitations |
| --- | --- | --- | --- |
| Effectiveness Against HPV Infection |  |  |  |
| A7  Markowitz LE, Liu G, Hariri S, Steinau M, Dunne EF, Unger ER. Prevalence of HPV after introduction of the vaccination program in the United States. Pediatrics 2016; 137: 1-9.  USA^1^ | NHANES - series of cross-sectional surveys conducted by the National Center for Health Statistics & CDC  NHANES surveys designed to be nationally representative of civilian, noninstitutionalized US population. Analyses for this study were limited to female NHANES participants aged 14 to 34 years with adequate self-collected cervicovaginal samples.  In 2003-2006, 3325 females aged 14 to 34 years were interviewed. Of those, 2649 submitted a sample, and 2587 samples were adequate for DNA typing. In 2009 to 2012, 2473 females aged 14 to 34 years were interviewed. Of those, 2070 submitted a sample, and 2061 were adequate for DNA typing. | Pre-vaccination period 2003-2006, post-vaccination period 2009-2012 | Strengths: Nationally representative sample from NHANES; adjusted prevalence ratios.  Limitations: vaccinated defined as >1 dose; self-reported vaccination status and self-collected specimens. |
| A8  Chow EP, Danielewski JA, Fehler G, et al. Human papillomavirus in young women with *Chlamydia trachomatis* infection 7 years after the Australian human papillomavirus vaccination programme: a cross-sectional study. Lancet Infect Dis 2015; 15: 1313-1323.  Australia^2^ | Repeat cross-sectional survey  Females aged ≤ 25 diagnosed with chlamydia at Melbourne Sexual Health Centre  Australian-born women in the pre-vaccination period (n=160). All women in the post-vaccination period (n=27) | Pre-vaccination 2004-2007, post-vaccination 2007-2014 | Strengths: clinician-collected specimens; prevalence ratios adjusted for potential confounders.  Limitations: Cross-sectional survey, measurement in chlamydia-positive population, self-reported vaccination status, “vaccinated” indicates ≥1 dose. |
| Impact on HPV Infection |  |  |  |
| A9  Dickson EL, Vogel RI, Luo X, Downs LS. Recent trends in type-specific HPV infection rates in the United States. Epidemiol Infect 2015; 143: 1042–1047.  USA^1^ | Repeat cross-sectional survey  Women aged 21-65 years who had HPV typing  n=220,914 | 2004 to 2011  Program dates not provided. | Not included in results because no HPV type-specific numeric estimates were reported. |
| A10  Dunne EF, Naleway A, Smith N, et al. Reduction in human papillomavirus vaccine type prevalence among young women screened for cervical cancer in an integrated US healthcare delivery system in 2007 and 2012-2013. J Infect Dis 2015; doi:10.1093/infdis/jiv342.  USA^1^ | Repeat cross-sectional survey  Women aged 20-29 undergoing routine cervical cancer screening at Kaiser Permanente Northwest.  2007-2012 (n=4138), 2012-2013 (n=4171) | 2007 to 2012, and 2012 to 2013. | Strengths: Included screened and insured population; vaccination status from electronic medical records, clinician-collected cervical specimens.  Limitations: Proportion of subjects vaccinated prior to sexual debut not reported. |
| A11  Kahn JA, Brown DR, Ding L, et al. Vaccine-type human papillomavirus and evidence of herd protection after vaccine introduction. Pediatrics 2012; 130: e249-256.  USA^1^ | Repeat cross-sectional survey  Females aged 13-26 in 2 primary care clinics in Cincinnati (1 hospital-based adolescent clinic; 1 community HC)  2006 to 2007 (n=368), 2009 to 2010 (n=409) | 2006 to 2007 and 2009 to 2010 | Strengths: Vaccination status from state registry system for 87% of sample; propensity score adjusted prevalence.  Limitations: proportion vaccinated prior to start of sexual activity not reported; high risk population; 13% self-reported vaccination status; clinician or self-sampled specimen- possible bias. |

| Reference #  Full Citation  Country | Data Source  Study Population  Sample Size | Data Collection Dates  Vaccination Program Comments | Strengths and Limitations |
| --- | --- | --- | --- |
| Impact on HPV Infection |  |  |  |
| A12  Markowitz LE, Hariri S, Lin C, et al. Reduction in human papillomavirus (HPV) prevalence among young women following HPV vaccine introduction in the United States, National Health and Nutrition Examination Surveys, 2003-2010. J Infect Dis 2013; 208: 385-393.  USA^1^ | Repeat cross-sectional survey  Females aged 14-19 in nationwide population sample: National Center for Health Statistics (NHANES) cross-sectional survey.  2003 to 2006 (n=736), post-vaccination 2007 to 2010 (n=358) | Pre-vaccination 2003 to 2006, post-vaccination 2007 to 2010 | Refer to A7 for updated data. |
| A13  Soderlund-Strand A, Uhnoo I, Dillner J. Change in population prevalence of human papillomavirus after initiation of vaccination: the high-throughput HPV monitoring study. Cancer Epidemiol Biomarkers Prev 2014; 23; 2757.  Sweden^3^ | Repeat cross-sectional survey  Females in Skane Region (Southern Sweden). Consecutive samples from the C. trachomatis screening analyzed for HPV DNA; large proportion of population participates in screening program (e.g. 23% of all 19 year old girls)  Analyzed 44,146 samples in 2008, 5,224 in 2012, and 5,815 in 2013 | Three study periods: 2008; 2012; and 2013 | Strengths: Large, population-based study; vaccination status from registry; clinician-collected specimen.  Limitations: Vaccination coverage for 18-22 year olds in pre-vaccine program era ranged from 2% - 17% (resultant misclassification would bias estimate towards the null); unadjusted estimates- no individual sexual behavior information. |

| Reference #  Full Citation  Country | Data Source  Study Population  Sample Size | Data Collection Dates  Vaccination Program Comments | Strengths and Limitations |
| --- | --- | --- | --- |
| Impact on HPV Infection |  |  |  |
| A14  Merckx M, Vanden Broeck D, Benoy I, Depuydt C, Weyers S, Arbyn M. Early effects of human papillomavirus vaccination in Belgium. Eur J Cancer Prev 2014; 24: 340-342.  Belgium^4^ | Cross-sectional study. Samples collected from AML Laboratory, Antwerp.  Women less than 25 years of age participating in opportunistic cervical cancer screening.  25,532 samples from females <25 years: 3395 samples from females <19 years, and 22,137 from females aged 19-25. | September 2009-January 2012.  Reimbursed in Belgium since 2007 for girls (12-15 years) with 4vHPV, extended to girls up to 18 years in 2008. From May 2008, reimbursement was extended to 2vHPV. A school-based programmatic approach based on the opt-out principle started from 2010 in the Flemish region, and 1 year later in the French-speaking region. | Strengths: large cervical screening population; clinician-collected specimen; Poisson regression trend analysis.  Limitations: no vaccination status information; no adjustment for potential confounding variables; unknown proportion vaccinated prior to start of sexual activity. |
| A15  Wilson AR, Welch RJ, Hashibe M, Greenwood J, Jackson B, She RC. Surveillance of human papilloma virus using reference laboratory data for the purpose of evaluating vaccine impact. Online J Public Health Inform 2014; 6: e194.  USA^1^ | Retrospective longitudinal data from HPV testing conducted at a national reference laboratory. Data from patient tests results archived in an electronic data warehouse were extracted for HPV results.  First observation for each patient per calendar year between 2004 and 2013.  735,437 total high-risk HPV results from 590,036 unique patients at 692 unique client sites in 48 U.S. states. | January 1, 2004 to June 1, 2013 | Not included in results because high-risk HPV genotypes were included in the publication, but not specifically 16/18 |

| Reference #  Full Citation  Country | Data Source  Study Population  Sample Size | Data Collection Dates  Vaccination Program Comments | Strengths and Limitations |
| --- | --- | --- | --- |
| Effectiveness Against Genital Warts |  |  |  |
| A16  Blomberg M, Dehlendorff C, Sand C, Kjaer SK. Dose-related differences in effectiveness of human papillomavirus vaccination against genital warts: a nationwide study of 550,000 young girls. Clin Infect Dis 2015; 61: 676-682.  Denmark^5^ | Linked population-based registries: CRVS, national health insurance register, prescription registry.  Females born during 1985–1999, for whom information on HPV vaccinations was retrieved.  GWs were diagnosed in 14,540 unvaccinated girls (2,217,764 person-years for 550,690 girls), in 370 girls after the first dose (111,005 person-years for 357,536 girls), in 261 girls after the second dose (178,319 person-years for 304,236 girls), and in 398 girls after all 3 doses (591,832 person-years for 212,551 girls). | October 2006 to December 2012 | Strengths: Large nationwide population-based cohort study. Fair follow-up time, limited loss to follow-up. Possibility to link outcome to individual vaccination status. Well recognized high quality of Nordic database. Fair attempt to limit confounding (IRR estimates stratified by birth cohort).  Limitations: Secondary data use, absence of case or vaccination status ascertainment. Possible lack of comparability between vaccinated and unvaccinated women in birth cohorts with high vaccine coverage rates. Potential remaining confounding due to the nature of the data source. |
| A17*  Dominiak-Felden G, Gobbo C, Simondon F. Evaluating the early benefit of quadrivalent HPV vaccine on genital warts in Belgium: a cohort study. PloS One 2015; 10: e0132404.  Belgium^4^ | National Union of Independent sick-funds (MLOZ) reimbursement database.  Females nationwide in Belgium. Jan 1990 to Dec 1997 birth cohort.  106,579 women were included in VE analysis, representing 369,881 person-years of follow-up. Over the study period, 274 first-time GWs episodes were recorded, 244 among unvaccinated women and 14 among women fully-vaccinated with quadrivalent HPV vaccine. | January 2006 to December 2013 | Strengths: Large reimbursement database analysis, allowing to link outcome occurrence to individual vaccination. Fair follow-up time. Use of specific surrogate for GW occurrence. Fair attempts to minimize confounding and biases.  Limitations: Absence of case or vaccine status ascertainment. Limited number of actual GW cases identified. Potential for remaining confounding due to the nature of the data source. |

| Reference #  Full Citation  Country | Data Source  Study Population  Sample Size | Data Collection Dates  Vaccination Program Comments | Strengths and Limitations |
| --- | --- | --- | --- |
| Effectiveness Against Genital Warts |  |  |  |
| A18  Herweijer E, Leval A, Ploner A, et al. Association of varying number of doses of quadrivalent human papillomavirus vaccine with incidence of condyloma. JAMA 2014; 311: 597-603.  Sweden^3^ | Swedish nationwide population-based health data registers: vaccination register, prescribed drug register (podophyllotoxin + imiquimod), patient register, and cause of death register  All females aged 10 to 24 years living in Sweden. All with condyloma prior to individual follow-up were excluded.  n = 1,045,165 followed up between 2006 and 2010 for HPV vaccination and first occurrence of condyloma | 2006-2010 | Strengths: Large nationwide population-based cohort study. Fair follow-up time, limited loss to follow-up. Possibility to link outcome to individual vaccination status. Acknowledged good quality of Nordic database. Fair attempt to limit confounding and biases.  Limitations: Secondary data use, absence of case or vaccination status ascertainment. Potential for remaining confounding due to the nature of the data source. |
| A19  Leval A, Herweijer E, Ploner A, et al. Quadrivalent human papillomavirus vaccine effectiveness: a Swedish national cohort study. J Natl Cancer Inst 2013; 105: 469-74  Sweden^3^ | Total population register, prescribed drug register, Swedish vaccination register  Women aged 10-44 years living in Sweden between 2006 and 2010. Individuals with GW before individual follow-up (n=15656) were excluded. Females who received the 2vHPV vaccine (n=1381) were excluded at vaccination. Women censored at time of death (n=3377), at their 45th birthday, or if they emigrated (data available only until 2002) (n=152,896)  >2.2 million between 2006 and 2010 | 2006-2010 | Strengths: Large nationwide population-based cohort study. Fair follow-up time, limited loss to follow-up. Possibility to link outcome to individual vaccination status. Acknowledged good quality of Nordic database. Fair attempt to limit confounding and biases.  Limitations: Secondary data use, absence of case or vaccination status ascertainment. Potential for confounding due to nature of data source. |

| Reference #  Full Citation  Country | Data Source  Study Population  Sample Size | Data Collection Dates  Vaccination Program Comments | Strengths and Limitations |
| --- | --- | --- | --- |
| Effectiveness Against Genital Warts |  |  |  |
| A20*  Smith LM, Strumpf EC, Kaufman JS, Lofters A, Schwandt M, Levesque LE. The early benefits of human papillomavirus vaccination on cervical dysplasia and anogenital warts. Pediatrics 2015; 135: e1131-1140.  Canada^6^ | Ontario Administrative health databases: registered person, health insurance plan, discharge abstract, same day surgery databases, and ambulatory care reporting system. Immunization records information system.  Girls in grade 8 before (2005/2006–2006/2007) and after (2007/2008–2008/2009) program implementation; identified from administrative databases.  Cohort 131,781 ineligible and 128,712 eligible girls (n = 260, 493); 2436 cases of dysplasia and 400 cases of anogenital warts. | Pre-vaccination period 2005-2007, post-vaccination period 2007-2009 | Strengths: Large cohort study conducted using population-based administrative databases analysis. Sensitive & specific HPV vaccination data. Large reimbursement database allows outcome occurrence to be linked to individual vaccination. Fair follow-up time.  Limitations: Use of 3 possible unascertained case definitions for GW, some of them being quite unspecific. Unclear exposure definition. Unusual overall methodology. Restricted study population (young cohort) with likely low generalizability. Potentially missing information from the girls vaccinated by private means. Limited number of actual GW cases identified. Potential for remaining bias or confounding. |
| A21  Blomberg M, Dehlendorff C, Munk C, Kjaer SK. Strongly decreased risk of genital warts after vaccination against human papillomavirus: nationwide follow-up of vaccinated and unvaccinated girls in Denmark. Clin Infect Dis 2013; 57: 929-934.  Denmark^5^ | CRVS, National Health Insurance Register (captures vaccination program), prescription registry (captures non-program vaccine purchases), National Patient Register (captures outcome), National Pathology Bank.  Females nationwide in Denmark. Only included birth cohorts 1989-1999 as they had a vaccine coverage rate of >10%. Excluded diagnoses prior to vaccine licensure date (vaccinated n=63, unvaccinated n=134) or during follow-up but before vaccination (n=334)  2006 denominator for all age-groups n=139561. 2012 denominator n=248403 | October 2006 to May 2012 | Strengths: Large nationwide population-based cohort study. Fair follow-up time, limited loss to follow-up. Possibility to link outcome to individual vaccination status. Acknowledged good quality of Nordic database. Fair attempt to limit confounding (IRR estimates stratified by birth cohort).  Limitations: Secondary data use, absence of case or vaccination status ascertainment. Possible lack of comparability between vaccinated and unvaccinated women in birth cohorts with high vaccine coverage rates. Potential confounding due to the nature of the data source. |

| Reference #  Full Citation  Country | Data Source  Study Population  Sample Size | Data Collection Dates  Vaccination Program Comments | Strengths and Limitations |
| --- | --- | --- | --- |
| Effectiveness Against Genital Warts |  |  |  |
| A22  Swedish KA. Prevention of anal condyloma with quadrivalent human papillomavirus vaccination of older men who have sex with men. PLoS One 2014; 9: e93393  USA^1^ | Medical charts of all eligible patients seen anorectal surgery practice (SEG) in New York City during 2007–2010 were screened for inclusion.  Study participants were recruited from a single anorectal surgery practice (SEG) in New York City that specializes in screening, diagnosis, and treatment of anorectal diseases. Majority of patients are MSM presenting with HPV related disease. Analysis included patients who were 26 years of age or older, HIV-negative, and self-identified MSM.  694 HIV-negative MSM were seen in the practice from 2007 to 2010. Based on chart review, 313 patients were ≥26 years of age without history of anal condyloma or with history of previously-treated anal condyloma recurrence-free for ≥12 months. | 2007-2010; Beginning in June 2006, the three-dose 4vHPV series was offered off-label to all HIV-negative MSM patients at each clinical visit to the anorectal surgery practice (SEG) in New York City | Strengths: Clinical diagnosis of GW. Individual vaccination status based on reimbursement data. Fair attempt to limit potential confounding and biases in analysis.  Limitations: Post-hoc retrospective analysis of a prospective cohort study. Single center. Likely limited external validity. Possible lack of comparability between vaccinated and unvaccinated subjects. Potential for remaining bias or confounding. |

| Reference #  Full Citation  Country | Data Source  Study Population  Sample Size | Data Collection Dates  Vaccination Program Comments | Strengths and Limitations |
| --- | --- | --- | --- |
| Impact on Genital Warts |  |  |  |
| A23  Ali H, Guy RJ, Wand H, et al. Decline in in-patient treatments of genital warts among young Australians following the national HPV vaccination program. BMC Infect Dis 2013; 13: 140.  Australia^2^ | In-patient treatment for genital warts in all private hospitals of Australia (Medicare – universal health insurance scheme)  Nationwide: Males and females. Between 2000 and 2011, a total of 2237 men aged 15-44 years underwent treatment for genital warts  Between 2000 and 2011, a total of 6014 women aged 15-44 years underwent in-patient treatment for vulvar/vaginal warts. Between 2000 and 2011, a total of 936 men aged 15-44 years underwent treatment for penile warts. | Pre-vaccination 2000-2007, post-vaccination 2007-2011 | Strengths: Large national reimbursement data base. Use of surrogate for GW, still seems a specific surrogate (patients treated under anesthesia for GW). Elaborated statistical methodology for an impact evaluation (time-series analysis), attempt to evaluate effect of some potential confounder for vaccine effect (stratification by age and gender), even if others remain.  Limitations: Inpatients only, private hospitals only. Ecological study, absence of external control, no possibility to distinguish incident vs prevalent cases, so potential for residual confounding. |
| A24  Ali H, Donovan B, Wand H. Genital warts in young Australians five years into national human papillomavirus vaccination programme: national surveillance data. BMJ 2013; 346; doi:10.1136/bmj.f2032.  Australia^2^ | National surveillance network, patients attending sexual health services (8 sexual health services dispersed across Australia) that could provide retrospective data to 2004..  Australia Nationwide: Males and females attending sentinel Health Facilities. | Pre-vaccination 2004-2007, post-vaccination 2007-2011 | Strengths: Large nationally representative sentinel surveillance network (8 sexual health centers and 2 large clinics), with resultant broad geographical representation. Clinical diagnosis of GW, incident GW only. Attempt to control for confounding (results stratified by age, gender and sexual orientation). External control (trends of Chlamydia within same period) and control population.  Limitations: Ecological study, potentially selected high risk population, potential for residual confounding. |

| Reference #  Full Citation  Country | Data Source  Study Population  Sample Size | Data Collection Dates  Vaccination Program Comments | Strengths and Limitations |
| --- | --- | --- | --- |
| Impact on Genital Warts |  |  |  |
| A25  Baandrup L, Blomberg M, Dehlendorff C, Sand C, Andersen KK, Kjaer S. Significant decrease in the incidence of genital warts in young Danish women after implementation of a national human papillomavirus vaccination program. Sex Transm Dis 2013; 40: 130-5  Denmark^5^ | Danish National Patient Register (nationwide registers)  Males and females who were hospitalized or had an outpatient consultation for GWs.  A total of 17,309 new cases of GWs were diagnosed in Denmark between July 2006 and July 2011, with most incident cases in women (57.5%). | 1995 to 2011 | Strengths: Large nationwide database analysis on hospital discharge data and outpatient consultations for GW, so fair external validity. Attempt to control for confounding (results stratified by age and gender). External control (other STI within study period). Attempts to identify incident GW cases only.  Limitations: Ecological study. GW cases identified on diagnosis codes without case ascertainment. Potential remaining biases due to the nature of the database or study design. |
| A26  Bauer HM, Wright G, Chow J. Evidence of human papillomavirus vaccine effectiveness in reducing genital warts: an analysis of California public family planning administrative claims data, 2007-2010. J Am J Public Health 2012; 102: 833-835.  USA^1^ | Claims data (visit or pharmacy dispensing) from the California  Family Planning Access Care and Treatment Program, which serves low-income individuals in California  Clients with GW documented (clinically or pharmaceutically) prior to 2007.  All females denominator n=3359368, numerator n=24436; all males denominator n=464064, numerator n=15084 | 2007 to 2010 | Strengths: Large local (California) study using a reimbursement database. Fair attempt to control for confounding (results stratified by age and gender).  Limitations: Ecological Study, trends could be explained by factors other than vaccination. Use of administrative reimbursement data : GW cases identified through ICD codes without case ascertainment, so potential for misclassification bias. Possible selected population (high risk, deprived) due to the type of data source, so likely not broadly representative. Possible additional bias or confounding due to the nature of the database and/or study design. Absence of external control. Intermediate level of vaccine uptake as well as intermediate decrease. Vaccine status not determined. |
| Reference #  Full Citation  Country | Data Source  Study Population  Sample Size | Data Collection Dates  Vaccination Program Comments | Strengths and Limitations |
| Impact on Genital Warts |  |  |  |
| A27  Chow EP, Read TR, Wigan R, et al. Ongoing decline in genital warts among young heterosexuals 7 years after the Australian human papillomavirus (HPV) vaccination programme. Sex Transm Infect 2015; 91: 214-219.  Australia^2^ | Health facility records from Melbourne Sexual Health Centre  Males and Females at first-time visit to the health center  A total of 81 939 patients were seen for the first time at MSHC from 1 July 2004 to 30 June 2014. Of these, 41 776 (51.0%) were born in Australia. Among the Australian-born patients, 4282 (10.2%) were diagnosed with genital warts (3037 men, 1242 women and 3 transgender). | Pre-vaccination 2004 to 2007, post-vaccination 2007 to 2014 | Strengths: Large retrospective study. Clinical diagnosis of GW. Long follow-up. Fair attempt to control for confounding (results stratified by age, gender and sexual orientation). Attempt to include control populations (MSM, women not targeted by vaccination program).  Limitations: A single sexual health center. May not be representative of the general population (geographic and demographic -high risk population). Possible self-reporting and recall bias. Ecological study. |
| A28  Donovan B, Franklin N, Guy R, et al. Quadrivalent human papillomavirus vaccination and trends in genital warts in Australia: analysis of national sentinel surveillance data. Lancet Infect Dis 2011; 11: 39-44.  Australia^2^ | National surveillance network, patients attending sexual health services (8 sexual health services dispersed across Australia)  Males and females nationwide  From January, 2004, to December, 2009, 112083 new patients (48 922 women and 63 161 men) attended one of the sexual health services in the surveillance network, and 9867 (9%) had a first diagnosis of genital warts. | 2004-2009; 2004-2007; 2007-2009 | Strengths: Large nationally representative sentinel surveillance network (8 sexual health centers and 2 large clinics). Clinical diagnosis of GW, incident GW only. Fair attempt to control for confounding results stratified by age, gender and sexual orientation. Attempt to include control populations.  Limitations: Ecological study, potentially selected high risk population, potential for residual confounding, absence of external control. |

| Reference #  Full Citation  Country | Data Source  Study Population  Sample Size | Data Collection Dates  Vaccination Program Comments | Strengths and Limitations |
| --- | --- | --- | --- |
| Impact on Genital Warts |  |  |  |
| A29  Fairley CK, Hocking JS, Gurrin LC, Chen MY, Donovan B, Bradshaw CS. Rapid decline in presentations of genital warts after the implementation of a national quadrivalent human papillomavirus vaccination programme for young women. Sex Transm Infect 2009; 85: 499-502  Australia^2^ | Health facility records from Melbourne Sexual Health Centre  Males and females attending Melbourne Sexual Health Centre.  Number of clients seen: during 2004-2007 n=28,093; in 2008 n=7962 | 2004-2008 | Strengths: Large ecological study using retrospective data. Clinical diagnosis of GW. Attempts to control for confounding (results stratified by age, gender and sexual orientation). Attempt to include a control population (MSM, women not targeted by vaccination program) and external control (primary genital herpes).  Limitations: A single sexual health center, not broadly representative population (geographic and demographic high-risk). Possible self-reporting and recall bias. |
| A30  Flagg EW, Schwartz R. Prevalence of anogenital warts among participants in private health plans in the United States, 2003-2010: potential impact of human papillomavirus vaccination. Am J Public Health 2013; 103: 1428-1435.  USA^1^ | Claims data (inpatient/outpatient visits or pharmacy dispensing) from large claims database (Truven Health Analytics). Data from ~100 health insurance plans.  Males and females aged 10 to 39 years with continuous insurance within a given year.  >64 million person-years of data were represented in the analysis | 2003-2010 | Strengths: Large study using a large nationally representative reimbursement database. Several type of data sources used (in-patient/outpatient, treatment), so likely good external validity. Fair attempt to control for confounding (results stratified by age, gender as well as other socio-demographic characteristics). Robust analytical methodology.  Limitations: Ecological study. GW identified through diagnoses codes and/or surrogate markers, so possible misclassification bias, still attempt to limit it by using an algorithm. No possibility to distinguish incident from prevalent GW, so possible duplicates. Short follow-up. Intermediate level of vaccine uptake as well as intermediate GW decrease. Potential for additional confounding and/or biases due to the nature of the database and/or study design |

| Reference #  Full Citation  Country | Data Source  Study Population  Sample Size | Data Collection Dates  Vaccination Program Comments | Strengths and Limitations |
| --- | --- | --- | --- |
| Impact on Genital Warts |  |  |  |
| A31  Harrison C, Britt H, Garland S, Conway L. Decreased management of genital warts in young women in Australian general practice post introduction of national HPV vaccination program: results from a nationally representative cross-sectional general practice study. PLoS One 2014; 9: e105967.  Australia^2^ | Nationwide health facility activity from general practice registers  Males and females attending general practitioners nationwide  1,175,879 patient encounters with 11,780 general practitioners | 2002-2006; 2008-2012 | Strengths: Large nationwide database analysis in general practice database. Long follow-up. Attempt to control for confounding (results stratified by age and gender). Attempt to include external controls (other STI).  Limitations: Ecological study. GW identified with diagnosis code. No possibility to distinguish between incident and prevalent cases. Possible other limitations/remaining confounding due to the nature of the database. |
| A32  Judlin P, Jacquard AC, Carcopino X, et al. Potential impact of the human papillomavirus vaccine on the incidence proportion of genital warts in French women (EFFICAE study): a multicentric prospective observational study. Sexual Health 2015; 13:49-54.  France^7^ | Prospective multicenter study from gynecologic practices. A total of 160 gynaecologists participated in T0 and 189 in T1. Prospective study with each gynaecologist enrolling all 15-26 year old women attending within a 4 month period.  French women aged 15-26 attending gynaecologists nationwide.  Patients aged 15-26 years old T0 n=39090; T1 n=45628 | 2008-2009; 2011-2012 | Strengths: Small but nationally representative prospective multi-centric cohort study. GW diagnosis assessed clinically, incident cases only. Attempt to limit confounding, presence of external control.  Limitations: Ecological study. Some elements differ between pre and post-vaccination periods (such as recruiting gynecologists, although still random selection each time, similar profile, etc). Limited follow-up. Limited vaccine coverage in study population. |

| Reference #  Full Citation  Country | Data Source  Study Population  Sample Size | Data Collection Dates  Vaccination Program Comments | Strengths and Limitations |
| --- | --- | --- | --- |
| Impact on Genital Warts |  |  |  |
| A33  Leval A, Herweijer E, Arnheim-Dahlstrom L, et al. Incidence of genital warts in Sweden before and after quadrivalent human papillomavirus vaccine availability. J Infect Dis 2012; 206: 860-866.  Sweden^3^ | National linked registers: population data from statistics in Sweden on genital warts episodes from the prescribed drug register and national patient register.  The entire population aged 10–44 years living in Sweden between 2006 and 2010.  The study population ranged from 4,167,770 individuals in 2006 to 4,190,658 in 2010. | 2006-2010 | Strengths: Large nationwide population-based cohort study. Several data sources used (inpatient/outpatient/ treatment reimbursement). Fair Attempt to control for confounding (results stratified by age and gender). Attempt to identify incident cases only.  Limitations: Ecological study. GW case identified though ICD codes or surrogate marker, possible misclassification bias. Absence of external control within study, but increasing trends in STI from another source described. Possible additional bias or confounding due to the nature of the database and/or study design. |
| A34  Liu B, Donovan B, Brotherton JM, Saville M, Kaldor JM. Genital warts and chlamydia in Australian women: comparison of national population-based surveys in 2001 and 2011. Sex Transm Infect 2014; 90: 532-537.  Australia^2^ | Nationwide phone survey (random digit dialing) derived from self-declaration  Sampling stratified by age to recruit women aged 21-30 years and 31-39 years in a ratio of 3:1, and by state or territory of residence.  2394 women were included in the 2011 survey. 88 (3.7%) reported a genital warts diagnosis, and 127 (5.3%) had at least one chlamydia diagnosis. 4874 women were surveyed in 2001. | 2001 vs. 2011 | Strengths: Small but nationally representative study. Sampling method for recruitment and interviewing methods seems robust and standardized. Attempts to reduce confounding. External control.  Limitations: Small number of GW cases identified. Ecological study. Self-reported GW cases, potential for misclassification bias and recall bias. Possible other limitations/remaining confounding due to the nature of the study. |

| Reference #  Full Citation  Country | Data Source  Study Population  Sample Size | Data Collection Dates  Vaccination Program Comments | Strengths and Limitations |
| --- | --- | --- | --- |
| Impact on Genital Warts |  |  |  |
| A35  Mikolajczyk RT, Kraut AA, Horn J, Schulze-Rath R, Garbe E. Changes in incidence of anogenital warts diagnoses after the introduction of human papillomavirus vaccination in Germany-an ecologic study. Sex Transm Dis 2013; 40: 28-31.  Germany | German pharmaco-epidemiological research database. Nationwide database from a large health insurance company (inpatient/outpatient/prescriptions)  Females aged between 12 and 19 nationwide  >6 million insured persons, 8% of the German population | 2005-2008; March 2007: The German Standing Vaccination Committee (STIKO) recommended HPV vaccination for girls between 12 and 17 years of age. It does not distinguish between the bivalent and quadrivalent vaccine, however the quadrivalent vaccine is used predominantly in Germany (almost 90% of doses) | Strengths: Large study using large nationally representative reimbursement database (in-patient/outpatient/ambulatory visit/ treatment). Likely good external validity. Attempt to control for confounding (results stratified by age, gender). Robust analytical methodology. Attempt to identify incident cases only.  Limitations: Ecological study. GW identified through diagnoses codes. Short follow-up. Potential for additional confounding and/or bias due to the nature of the database and/or study design. |
| A36  Oliphant J, Perkins N. Impact of the human papillomavirus (HPV) vaccine on genital wart diagnoses at Auckland Sexual Health Services. New Zealand Medical Journal 2011; 124: 51-8.  New Zealand^8^ | New patients attending Auckland Sexual Health Services (ASHS).  New patients to ASHC in the study period. Women aged between 20 and 21 were excluded from analyses as some but not all would have been eligible to receive the vaccine.  40,793 new patients attended the ASHS between 2007 and June 2010 and genital warts were diagnosed in 3125 (7.7%). Genital warts were diagnosed in 9.2% of new patients in 2007 decreasing to 6.6% for the first 6 months of 2010. | 2007-2008; 2009-2010 | Strengths: Clinical diagnosis of GW. Fair attempt to control for confounding (results stratified by age and gender).  Limitations: Medium-sized, local monocentric retrospective chart database analysis. Selected (high risk) population, so likely limited generalizability. Ecologic study. No external control. Potential for additional confounding and/or biases due to the nature of the database and/or study design. Intermediate level of vaccine uptake as well as intermediate decrease. |

| Reference #  Full Citation  Country | Data Source  Study Population  Sample Size | Data Collection Dates  Vaccination Program Comments | Strengths and Limitations |
| --- | --- | --- | --- |
| Impact on Genital Warts |  |  |  |
| A37  Read TR, Hocking JS, Chen MY, Donovan B, Bradshaw CS, Fairley CK. The near disappearance of genital warts in young women 4 years after commencing a national human papillomavirus (HPV) vaccination programme. Sex Transm Infect 2011; 87: 544-547.  Australia^2^ | Health facility records from Melbourne Sexual Health Centre  New patients attending MSHC between 1 July 2004 and 30 June 2011  From 1 July 2004 to 30 June 2011, 52 454 new patients were seen at MSHC and 5021 (9.6%, 95% CI 9.3% to 9.8%) were diagnosed  with GWs. | 2004-2007; 2007-2011 | Strengths: Large study using retrospective data. Clinical diagnosis of GW. Long follow-up. Attempt to control for confounding (results stratified by age, gender and sexual orientation). Attempt to include a control population (MSM, women non-targeted by vaccination program).  Limitations: A single sexual health centre; may not broadly represent the general population (geographic and demographic high risk population). Possible self-reporting and recall bias. Ecological study. |
| A38  Sando N, Kofoed K, Zachariae C, Fouchard J. A reduced national incidence of anogenital warts in young Danish men and women after introduction of a national quadrivalent human papillomavirus vaccination programme for young women--an ecological study. Acta Dermato-venereologica 2014; 94: 288-292.  Denmark^5^ | GW treatment prescription from Register of Medical Products statistics combined with inpatient/outpatient data from the National Patient Register  Males and females  The entire population of young people aged 15–34 in Denmark in 2001–2011 was included in this study | 2001-2008; 2008-2011 | Strengths: Large nationwide population-based database analysis on hospitals discharge (public + private) and reimbursement of specific treatment of GW. Attempt to control for confounding (results stratified by age and gender). External control (other STI).  Limitations: Ecological study. GW case identified though ICD codes or surrogate marker. Possible under-evaluation of GW cases, but unlikely to affect before/after comparison. Possible other limitations/remaining confounding due to the nature of the study. |

| Reference #  Full Citation  Country | Data Source  Study Population  Sample Size | Data Collection Dates  Vaccination Program Comments | Strengths and Limitations |
| --- | --- | --- | --- |
| Impact on Genital Warts |  |  |  |
| A39  Smith MA, Liu B, McIntyre P, Menzies R, Dey A, Canfell K. Fall in genital warts diagnoses in the general and indigenous Australian population following implementation of a national human papillomavirus vaccination program: Analysis of routinely collected national hospital data. J Infect Dis 2015; 211: 91-99.  Australia^2^ | Hospital admissions for GW from National Hospital Morbidity Database (public + private hospitals)  All hospital admissions between 1999–2011 involving a diagnosis of genital warts 39 350 admissions involving a diagnosis of genital warts (24 811 in females; 14 539 in males) were recorded in the NHMD during July 1999–June 2011 | 2004-2007; 2008-2011 | Strengths: Large nationwide database analysis of all private and public hospitals. Attempt to control for confounding (results stratified by age, gender, and other socio-demographic characteristics).  Limitations: Ecologic study. GW identified based on ICD codes, no differentiation between prevalent and incident cases. No control group but description of concurrent STI disease trends from other sources. Potential for additional confounding and biases, due to the nature of the data source and study type. |
| A40  Wilson N, Morgan J, Baker MG. Evidence for effectiveness of a national HPV vaccination programme: national prescription data from New Zealand. Sex Transm Infect 2014; 90: 103.  New Zealand^8^ | National prescription data from the government's pharmaceutical purchasing agency  Prescriptions nationwide  Podopyllum: pre-vaccination range 5375-5449, post-vaccination 5266; Imiquimod: n=4025 in 2009-2010, n=3138 in 2011/2012 | 1998/1999 to 2011/2012 | Strengths: Large nationwide reimbursement database analysis.  Limitations: Surrogate used for GW diagnosis. No possibility to control for any confounding. Ecologic study. No external control. Potential for confounding and/or biases due to the nature of the database and/or study design. |
| A41  Nsouli-Maktabi H, Ludwig SL, Yerubandi UD, Gaydos JC. Incidence of genital warts among male and female service members aged 17 years and older before and after the introduction of the quadrivalent human papillomavirus vaccine. MSMR 2013; 20: 17-20.  USA^1^ | Healthcare encounters with GW diagnosis within the Defense Medical Surveillance System (DMSS).  U.S. service members throughout their military service  1,544,029 in 2000 to 1,440,362 in 2012 | January 2000 to December 2012; HPV4 vaccine licensed for use in women aged 9-26 years in 2006 and for men 9-26 years in 2009. Since its licensure, 4vHPV vaccine has been added to U.S. civilian and Department of Defense (DoD) immunization programs. | Strengths: Large reimbursement database analysis, including all types of medical encounter. Attempt to control for confounding (stratified by age, gender). Attempt to identify incident cases only. Long follow-up.  Limitations: Possibly selected population. No detailed result on external control, description of other STI trends in study population briefly mentioned. Ecologic study. GW identified based on ICD codes without ascertainment. Potential for confounding and/or biases due to the nature of the database and/or study design. |

| Reference #  Full Citation  Country | Data Source  Study Population  Sample Size | Data Collection Dates  Vaccination Program Comments | Strengths and Limitations |
| --- | --- | --- | --- |
| Impact on Genital Warts |  |  |  |
| A42  Bollerup S, Baldur-Felstov B, Blomberg M, Baandrup L, Dehlendorff C, Kjaer SK. Significant reduction in the incidence of genital warts in young men 5 years into the Danish HPV vaccination program for girls and women. Sex Transm Dis 2016; 43: 238-242.  Denmark^5^ | GW treatment prescriptions from Register of Medical Products statistics combined  With inpatient/outpatient data from the National Patient Register Population data from Statistics Denmark.  Nationwide. Both men and women were included.  Person years by sex and age (2008; 2013): Males age 12-15 (145892; 139869), 16-17 (69286; 73554), 18-19 (66040; 74553), 20-21 (63382; 74602), 22-25 (121812; 142526), 26-29 (129732; 128669), 30-35 (224331; 199287), 36+ (1484539; 1549231).  Females age 12-15 (138383; 133404), 16-17 (65759; 69558), 18-19 (62656; 70966), 20-21 (60388; 71475), 22-25 (118832; 138026), 26-29 (129374; 126549), 30-35 (222768; 197872), 36+ (1576453; 1638571) | 2006-2013 | Strengths: Large nationwide population-based database analysis on hospitals discharge (public and private) and reimbursement of specific 1st line treatment of GW. Long follow-up. Attempt to control for confounding (results stratified by age and gender). External control (other STI).  Limitations: Ecological study. GW case identified though ICD codes or surrogate marker. Possible under-evaluation of GW cases, but unlikely to affect before/after comparison. Possible remaining confounding due to the nature of the study. |

| Reference #  Full Citation  Country | Data Source  Study Population  Sample Size | Data Collection Dates  Vaccination Program Comments | Strengths and Limitations |
| --- | --- | --- | --- |
| Impact on Genital Warts |  |  |  |
| A43  Smith MA, Liu B, McIntyre P. Trends in genital warts by socioeconomic status after the introduction of the national HPV vaccination program in Australia: Analysis of national hospital data. BMC Infect Dis 2016; 16: 52.  Australia^2^ | Hospital admissions for GW- from National Hospital Morbidity Database (public + private hospitals) Population estimates from the Australian Bureau of Statistics.  Nationwide. Both men and women were included.  Admissions 2004-2011 involving a diagnosis of GW by sex, and socioeconomic status [more disadvantaged=MD; less disadvantaged=LD] (n): Females age 10-19 MD n=947 LD=746; age 20-29 MD n=2061 LD n=2610; age 30-39 MD n=1034 LD n=1308. Males age 10-19 MD n=118 LD n=144; age 20-29 n=956 LD n=1636; age 30-39 MD n=654 LD n=1146. | 2004-2011 | Strengths: Large nationwide database analysis of all private and public hospitals. Attempt to control for confounding (results stratified by age, gender, and other socio-demographic characteristics).  Limitations: Ecologic study. GW identified based on ICD codes without ascertainment, no differentiation between prevalent and incident cases. No control group but description of concurrent STI disease trends from other sources. Potential for additional confounding and biases, due to the nature of the data source and study type. |

| Reference #  Full Citation  Country | Data Source  Study Population  Sample Size | Data Collection Dates  Vaccination Program Comments | Strengths and Limitations |
| --- | --- | --- | --- |
| Impact on Genital Warts | |  |  |
| A17*  Dominiak-Felden G, Gobbo C, Simondon F. Evaluating the early benefit of quadrivalent HPV vaccine on genital warts in Belgium: A cohort study. PloS One 2015; 10: e0132404.  Belgium^4^ | Large Belgian reimbursement database (MLOZ sick fund)  Males and females nationwide in Belgium.  VI analyses included 907,047 individuals in 2006, increasing to 1,284,493 individuals in 2013, providing 9,223,384 person-years of follow-up. During this period, 8,090 new GW episodes (4,533 for women and 3,557 for men) were recorded. | 2006; 2007-2008; 2009-2013 | Strengths: Large nationally representative reimbursement database analysis. Fair follow-up time. Use of specific surrogate for GW occurrence. Attempt to use incident cases only. Attempts to minimize confounding and biases.  Limitations: Absence of case ascertainment. Absence of external control on same database, but description of STI trends in study population. Potential for remaining confounding due to the nature of the data source. |
| A20*  Smith LM, Strumpf EC, Kaufman JS, Lofters A, Schwandt M, Levesque LE. The early benefits of human papillomavirus vaccination on cervical dysplasia and anogenital warts. Pediatrics 2015; 135: e1131-e1140.  Canada^6^ | Ontario's administrative health database (inpatient/outpatient visits).  Girls in grade 8 before (2005/2006–2006/2007) and after 2007/2008–2008/2009) program implementation was identified from administrative health databases.  The cohort comprised 131 781 ineligible and 128 712 eligible girls (total n = 260 493), with 2436 cases of dysplasia and 400 cases of anogenital warts. | Pre-vaccination period 2005-2007, post-vaccination period 2007-2009 | Strengths: Large cohort study conducted using population-based administrative databases analysis. Sensitive & specific HPV vaccination data. Large reimbursement database allows outcome occurrence to be linked to individual vaccination. Fair follow-up time.  Limitations: Use of 3 possible unascertained case definitions for GW, some of them being quite unspecific. Unclear exposure definition. Unusual overall methodology. Restricted study population (young cohort) with likely low generalizability. Potentially missing information from the girls vaccinated by private means. Limited number of actual GW cases identified. Potential for remaining bias or confounding. |

| Reference #  Full Citation  Country | Data Source  Study Population  Sample Size | Data Collection Dates  Vaccination Program Comments | Strengths and Limitations |
| --- | --- | --- | --- |
| Effectiveness Against Cervical Lesions | |  |  |
| A20*  Smith LM, Strumpf EC, Kaufman JS, Lofters A, Schwandt M, Levesque LE. The early benefits of human papillomavirus vaccination on cervical dysplasia and anogenital warts. Pediatrics 2015; 135: e1131-e1140.  Canada^6^ | Administrative health databases in Ontario: registered person database, Ontario health insurance plan database, discharge abstract database, same day surgery database, and ambulatory care reporting system. Immunization records information system.  Girls in grade 8 before (2005/2006–2006/2007) and after 2007/2008–2008/2009) program implementation were identified from administrative health databases.  The cohort comprised 131 781 ineligible and 128 712 eligible girls (total n = 260 493), with 2436 cases of dysplasia and 400 cases of anogenital warts. | Pre-vaccination period 2005-2007, post-vaccination period 2007-2009 | Strengths: Large cohort study conducted using population-based administrative databases analysis. Sensitive & specific HPV vaccination data. Large reimbursement database allows outcome occurrence to be linked to individual vaccination. Fair follow-up time.  Limitations: Use of 3 possible unascertained case definitions for GW, some of them being quite unspecific. Unclear exposure definition. Unusual overall methodology. Restricted study population (young cohort) with likely low generalizability. Potentially missing information from the girls vaccinated by private means. Limited number of actual GW cases identified. Potential for remaining bias or confounding. |

| Reference #  Full Citation  Country | Data Source  Study Population  Sample Size | Data Collection Dates  Vaccination Program Comments | Strengths and Limitations |
| --- | --- | --- | --- |
| Effectiveness Against Cervical Lesions | |  |  |
| A44  Brotherton JML, Malloy M, Budd AC, Saville M, Drennan KT, Gertig DM. Effectiveness of less than three doses of quadrivalent human papillomavirus vaccine against cervical intraepithelial neoplasia when administered using a standard dose spacing schedule: Observational cohort of young women in Australia. Papillomavirus Research (PVR) 2015; doi:10.1016/j.pvr.2015.05.005.  Australia^2^ | Deterministic data linkage was undertaken between the Victorian Cervical Cytology Registry and National HPV Vaccination Program Register to determine quadrivalent HPV vaccination status and incidence of cervical pathology.  Vaccine eligible women (aged 26 years or younger in 2007) screened in Victoria, Australia between April 2007 and December 2011. Registers are opt-out. Analysis only considered a cohort of women with a screening history, who had a Pap test recorded on the VCCR during the study period.  Number of women/women-doses: unvaccinated (n=133055); vaccinated adjusted - before (n=60934) & after (n=95489); 1 dose before (n=6938) & after (n=13720); 2 doses before (n=8638) & after (n=18863); 1 or 2 doses before (n=15576) & after (n=32583); 3 doses before (n=45358) & after (n=62906) | April 2007 to December 2011 | Strengths: Population-based analysis of the first 5 years of screening data; measures are adjusted hazard ratios; vaccination status and case determination from registry data.  Limitations: Follow-up average 2.9 years; females screened at age <20 years suggests a higher risk population and may not be generalizable to overall population. |

| Reference #  Full Citation  Country | Data Source  Study Population  Sample Size | Data Collection Dates  Vaccination Program Comments | Strengths and Limitations |
| --- | --- | --- | --- |
| Effectiveness Against Cervical Lesions | |  |  |
| A45  Crowe E, Pandeya N, Brotherton JM, et al. Effectiveness of quadrivalent human papillomavirus vaccine for the prevention of cervical abnormalities: case-control study nested within a population based screening programme in Australia. BMJ (Clin Res ed) 2014; 348: g1458.  Australia^2^ | Case-control study. Population registers in Queensland Health Pap Smear Register linked with Queensland vaccination administration system    Included all female Queensland residents who attended for their first ever cervical smear test between 1 April 2007 and 31 March 2011 and who had been eligible for HPV vaccination during the nationally funded catch-up programme (those born between July 1980 and July 1997). See exclusion criteria in text.  High grade cases (HGANY) were women with histologically confirmed high grade cervical abnormalities (n=1062) and “other cases” (OTHER) were women with any other abnormality at cytology or histology (n=10 887). Controls were women with normal cytology (n=96 404). | April 2007 to March 2011 | Strengths: Population-based analysis (n=96,404) with adjusted odds-ratio measures; vaccination and case status data obtained from linked administrative datasets.  Limitations: Follow-up time: low-grade/other cases: 1.8 years, high-grade cases: 2.1 years, controls: 2.2 years; small sample (n=6) of high grade cases with three doses among 11-14 year olds; misclassification of dose number may have occurred-vaccination database inaccuracies noted. |

| Reference #  Full Citation  Country | Data Source  Study Population  Sample Size | Data Collection Dates  Vaccination Program Comments | Strengths and Limitations |
| --- | --- | --- | --- |
| Effectiveness Against Cervical Lesions | |  |  |
| A46  Gertig DM, Brotherton JM, Budd AC, Drennan K, Chappell G, Saville AM. Impact of a population-based HPV vaccination program on cervical abnormalities: a data linkage study. BMC Med 2013; 11: 227.  Australia^2^ | Retrospective cohort. Victorian Cervical Cytology Registry linked to National HPV Vaccination Program Register.  VCC Register includes all women residents in Victoria, Australia (<1% opt out). NHVP Register records of HPV vaccination doses administered nationwide - believed to be virtually complete.  Number of women-doses: unvaccinated (n=15192), 1 dose (n=2568), 2 doses (n=3412), 1 or 2 doses (n=2568), 3 doses (n=21199), 4 doses (n=15192). | April 2007 to December 2011 | Strengths: Population-based analysis for first five years; n=24,871; Estimates are adjusted hazard ratios; vaccination and case status determined from registry data.  Limitations: Average follow-up time 1.5 years; females screened at age 12-17 years suggests higher risk population and may not be generalizable to overall population. |

| Reference #  Full Citation  Country | Data Source  Study Population  Sample Size | Data Collection Dates  Vaccination Program Comments | Strengths and Limitations |
| --- | --- | --- | --- |
| Effectiveness Against Cervical Lesions | |  |  |
| A47  Mahmud SM, Kliewer EV, Lambert P, Bozat-Emre S, Demers AA. Effectiveness of the quadrivalent human papillomavirus vaccine against cervical dysplasia in Manitoba, Canada. J Clin Onc 2014; 32: 438-43.  Canada | Linked longitudinal databases of data collected in Manitoba including the Manitoba Immunization Monitoring System and the Cervical Cancer Screening Registry.  Females 15 years old who resided in Manitoba and registered with MH between August 2006 and March 2010 (the enrollment period). All participants were required to have >3 years of insurance coverage before enrollment. Majority of participants vaccinated privately. Matched cohort: 1 vaccine recipient matched with 3 unvaccinated controls.    Vaccinated (n=3,541), unvaccinated (n=9,594) | August 2006 to March 2010; Introduced in August 2006; Manitoba has a provincial immunization program providing free, publicly-funded HPV vaccine to those who are eligible. | Strengths: Vaccinated (n=3,541), unvaccinated (n=9,594); adjusted vaccine effectiveness measures; vaccination status and cytology outcomes identified from population-based registries/databases.  Limitations: vaccination outside school-based program; vaccinated defined as receipt of any dose. |
| A48  Baldur-Felskov B, Dehlendorff C, Munk C, Kjaer SK. Early impact of human papillomavirus vaccination on cervical neoplasia--nationwide follow-up of young Danish women. J Natl Cancer Inst 2014; 106: djt460.  Denmark^5^ | Registers: CRVS linked with NHS registry and Danish National Prescription registry  Nationwide. Identified all girls and women born in Denmark. Only included birth cohorts with vaccination coverage greater than 10%, corresponding to birth cohorts 1989-1999. Excluded women who had received the bivalent vaccine (n=148). There were two comparison groups: Unvaccinated vs. vaccinated.  399244 Danish women were in the birth cohorts 1989 to 1999. | October 2006 to March 2012 | Strengths: Nationwide study (n=399,244 females); comparison to contemporaneous unvaccinated; vaccination status and lesions determined from national registers.  Limitations: Small n for CIN3+ 1993-94 cohort; Vaccinated defined as receipt of one or more dose; atypia+ (atypical cytology or worse- not histological endpoint); no control for potential confounders; small number of screened females (126) in 1995-96 cohort. |

| Reference #  Full Citation  Country | Data Source  Study Population  Sample Size | Data Collection Dates  Vaccination Program Comments | Strengths and Limitations |
| --- | --- | --- | --- |
| Effectiveness Against Cervical Lesions | |  |  |
| A49  Hariri S, Bennett NM, Niccolai LM, et al. Reduction in HPV 16/18-associated high grade cervical lesions following HPV vaccine introduction in the United States - 2008-2012. Vaccine 2015; 33: 1608-1613.  USA^1^ | HPV-IMPACT Project: Population-based sentinel surveillance system to monitor HPV vaccine impact on type-specific CIN2+. Vaccination and cervical cancer screening information was retrieved. Archived diagnostic specimens were obtained from reporting laboratories for HPV DNA typing. Cases were identified through electronic health record searches or manual chart reviews using all classification systems and terminology used for CIN2+ during the monitoring period. Population information source was 2010 U.S. Census data.  Females >18 years, residing in five catchment areas in California, Connecticut, New York, Oregon, and Tennessee.  The total population of females > 18 years ranges from 230,000 to 330,000 in each participating site. From 2008 to 2012, 7346 women who were age-eligible for vaccination (aged ≤26 years in 2006) were reported to HPV-IMPACT. Specimens were tested for 4693 women, and valid HPV DNA results were obtained for 4678 (99.7%). HPV DNA was detected in 4575(97.8%) | 2008 to 2012 | Strengths: Population-based surveillance in 5 catchment areas in US; CIN2+ n=549 vaccinated (≥1 dose before >12 months prior to pap test) 152 of whom had CIN3/AIS, 1274 not vaccinated 427 of whom had CIN3/AIS; adjusted prevalence ratios, medical record confirmed CIN diagnoses, HPV typed & only included 16/18-related lesions.  Limitations: Comparison is unvaccinated vs vaccinated (any dose); vaccination status obtained from multiple sources; 2215 lesions not included in analysis because vaccination status unknown. |

| Reference #  Full Citation  Country | Data Source  Study Population  Sample Size | Data Collection Dates  Vaccination Program Comments | Strengths and Limitations |
| --- | --- | --- | --- |
| Effectiveness Against Cervical Lesions | |  |  |
| A50  Herweijer E, Sundstrom K, Ploner A, Uhnoo I, Sparen P, Arnheim-Dahlstrom L. Quadrivalent HPV vaccine effectiveness against high-grade cervical lesions by age at vaccination: A population-based study. Int J Cancer 2016. Epub Date 2016/02/10.  Sweden^3^ | Data collected using Swedish population-based health data registers. 4vHPV and 2vHPV vaccination status collected using the Swedish HPV Vaccination register, National Vaccination register, and Prescribed Drug register. The National Swedish Cervical Screening Registry (NKCx) includes information on all pap-smears taken in Sweden as part of the cervical screening program but also all external to the program. NKCx was used to obtain information on invitation to organized cervical screening, cytological pap-smear results, and histologically confirmed diagnosis of the outcome. Cervical cancer cases and histologically confirmed high-grade cervical lesions collected from the Swedish Cancer register. Death and Migration register.  Nationwide cohort of women in Sweden 2006-2013 and aged 13-29. Start of follow-up was 1 Jan 2006 or 13th birthday. End of follow up was the end of the study (31/12/2013) or any of the following: diagnosis of outcome, 30th birthday, death, emigration, or 2vHPV vaccination.  Total number of women in cohort n=1,333,691 | January 1 2006 to 31 December 2013 | Strengths: All resident females 13-29 years old at vaccination initiation (n=1,333,691) females; follow-up to 8 years; adjusted incidence ratios from Poisson regression incorporating 6-month lag time; vaccination status and lesion diagnosis determined from national registers.  Limitations: Small n for CIN3+<17 years old; comparison group is partially vaccinated and unvaccinated. |

| Reference #  Full Citation  Country | Data Source  Study Population  Sample Size | Data Collection Dates  Vaccination Program Comments | Strengths and Limitations |
| --- | --- | --- | --- |
| Impact on Cervical Lesions |  |  |  |
| A51  Baldur-Felskov B, Dehlendorff C, Junge J, Munk C, Kjaer SK. Incidence of cervical lesions in Danish women before and after implementation of a national HPV vaccination program. Cancer Causes Control 2014; 25: 915-922.  Denmark^5^ | Nationwide pathology data bank (register)    Nationwide  In year 2000, the total female population > 12 years was 2,302,441 of which 1,194,770 were screened. This increased year on year and in 2012 the total and screened populations were 2,431,726 and 1,202,178 respectively. | Pre-vaccination period 2000-2005, transition period 2005-2010, post-vaccination period 2010-2013 | Strengths: Nationwide study; lesion diagnoses determined from national registers.  Limitations: Atypia+ (atypical cytology or worse, instead of a specific histopathological endpoint); not adjusted for vaccination status. |
| A52  Baldur-Felskov B, Munk C, Nielsen T S, et al. Trends in the incidence of cervical cancer and severe precancerous lesions in Denmark, 1997-2012. Cancer Causes Control 2015; 26: 1105-1116.  Denmark^5^ | Nationwide pathology data bank (register)  Nationwide  5,927 cases of cervical cancer were diagnosed in Denmark during 1997–2011 | 1997-2011 | Strengths: Nationwide study; lesion diagnoses determined from national registers.  Limitations: Atypia+ (atypical cytology or worse, instead of a specific histopathological endpoint); not adjusted for vaccination status. |
| A53  Brotherton JM, Fridman M, May CL, Chappell G, Saville AM, Gertig DM. Early effect of the HPV vaccination programme on cervical abnormalities in Victoria, Australia: an ecological study. Lancet 2011; 377: 2085-2092.  Australia^2^ | Victorian Cervical Cytology Registry.  Victoria  Number of women screened before vaccination (n=2418184), and after vaccination (n=1564840) | 2003-2007; 2007-2009 | Refer to A44 for updated data. |

| Reference #  Full Citation  Country | Data Source  Study Population  Sample Size | Data Collection Dates  Vaccination Program Comments | Strengths and Limitations |
| --- | --- | --- | --- |
| Impact on Cervical Lesions |  |  |  |
| A54  Brotherton JML, May CL, Chappell G, Gertig DM. Human papillomavirus vaccination is changing the epidemiology of high-grade cervical lesions in Australia. Cancer Causes Control 2015; 26: 953–954.  Australia^2^ | Victorian Cervical Cytology Registry.  Victoria | Comparisons were made between 2006 and 2013 for age <20, and 2008 and 2013 for age 20-24 | Refer to A44 (effectiveness study of similar population). |
| A55  Hariri S, Johnson ML, Bennett NM, et al. Population-based trends in high-grade cervical lesions in the early human papillomavirus vaccine era in the United States. Cancer 2015; 121: 2775-81.  USA^1^ | Sentinel system - population-based laboratory surveillance  Cases were reported to the HPV-IMPACT Project, a sentinel system for monitoring the population impact of HPV vaccine: population-based laboratory surveillance of 18 to 29 year old residents of California, Connecticut, New York, or Oregon  9119 cases of CIN2+ | 2008 to 2012 | Strengths: Data from population-based surveillance. Study demonstrates the challenges in interpreting ecological impacts of vaccination during time of change in cervical screening recommendations. Study shows the relationship between declining rates of CIN2+ and declining rates of cervical screening in women <21 years of age.  Limitations: Refer to A49 for summary of HPV-IMPACT results of vaccine effectiveness (rather than this ecologic analysis), which is summarized further in the manuscript figure. |
| A56  Jemal A, Simard E, Dorell C, et al. Annual report to the nation on the status of cancer, 1975-2009, featuring the burden and trends in human papillomavirus (HPV)-associated cancers and HPV vaccination coverage levels. J Natl Cancer Inst 2013; 105: 175-201.  USA^1^ | Registers and national surveys (CDC, NCI and NAACR. Population estimates from the census)  Nationwide: registers and national surveillance from CDC, NCI, and NAACCR sources. Population estimates from the census.  11388 cases of cancer of the cervix in 2009 | 1992 to 2009 | Provided cervical cancer incidence rates, without information on cervical precancerous lesions; no other data relevant to impact or effectiveness. |

| Reference #  Full Citation  Country | Data Source  Study Population  Sample Size | Data Collection Dates  Vaccination Program Comments | Strengths and Limitations |
| --- | --- | --- | --- |
| Impact on Cervical Lesions |  |  |  |
| A57  Niccolai LM, Julian PJ, Meek JI, McBride V, Hadler JL, Sosa LE. Declining rates of high-grade cervical lesions in young women in Connecticut, 2008-2011. Cancer Epidemiol Biomarkers Prev 2013; 22: 1446-1450.  USA^1^ | Surveillance data: all 34 pathology labs in state. Linked to surveillance data.  Statewide in Connecticut: surveillance data from all 34 pathology labs in state, linked to census data.  Total of 411624 women. Age 21 (n=87507), 25 (n=106159), 30 (n=104194), 35 (n=113764) | 2008 to 2011 | Not included in results. Connecticut is in CDC’s HPV-IMPACT program; refer to Hariri A55 for more thorough analysis. |
| A58  Ogilvie GS, Naus M, Money DM, et al. Reduction in cervical intraepithelial neoplasia in young women in British Columbia after introduction of the HPV vaccine: An ecological analysis. Int J Cancer 2015; 137: 1931-1937.  Canada | Cervical cancer screening program, population registries  British Columbia cervical cancer screening program, immunization registries  Population of 4.5 million | 2004 to 2012  In Canada, all programs use the 4vHPV vaccine and target pre-adolescent girls between Grades 4 and Grade 8. British Columbia, Canada’s western-most province, launched its HPV vaccine program for girls in September 2008. The program is delivered in Grade 6, but also had a three year catch up program in Grade 9 which ended in June 2011. | Strengths: Population based registries.  Limitations: Small n=10 cases of CIN2+ among vaccine-eligible females 15-17 years old. Authors also report 90% reduction in group <18 years of age but it is unclear how these estimates differ from the estimate of 69% also reported. Ecological analysis – many potential confounding factors uncontrolled and individual-level vaccination status unknown. Pap screening guidelines changed in 2011, resulting in 30-50% reduction in cytology specimens. This timing also corresponding with “vaccine era”, overlapping with years that birth cohorts became eligible for vaccination (2010-2012). During the vaccine era, a reduced number of cervical specimens were collected in comparison to the prevaccine era, possibly reflecting a population at higher risk of CIN2+ in the vaccine era. |

*Reference is repeated in another section, but its number at first mention is maintained.

VE, vaccine effectiveness; GW, genital warts; qHPV = 4vHPV.

**Vaccination Program Details:**

^1^USA: 4vHPV vaccine was licensed for use in the United States in June of 2006 and the Advisory Committee on Immunization Practices recommended routine vaccination with 3 doses of 4vHPV vaccine for females aged 11 or 12 years and catch-up vaccination for those aged 13 through 26 years. In October 2009, this recommendation was updated to include either HPV vaccine and ACIP stated it may be given to males aged 9 to 26 years then (in 2011) routinely recommended in aged 11 and 12 males, with catch-up in males aged 13 to 21 years.

^2^Australia: Vaccination Program from April 2007 through December 2009, school-based delivery strategy was used to offer free HPV vaccination to girls aged 12–18 years, and from July 2007 through December 2009, general practitioners and other community providers offered free vaccination to women aged ≤26 years. Since 2009, routine HPV vaccination has continued for girls in the first year of high-school (age 12–13 years) as part of the National Immunization Schedule. Program was expanded to include boys aged 12–13 years in February, 2013, with a catch up for ages 14–15 years up to December 2014.

^3^Sweden: Opportunistic vaccination began in October 2006. In Sweden, partially subsidized HPV vaccination was made available for girls ages 13-17 between May 2007-2011. Girls and women outside this target age range could receive the vaccine but were not eligible for reimbursement. An organized school-based vaccination program using the 4vHPV vaccine started in 2012 where girls ages 10-12 are vaccinated with 3 doses of the 4vHPV vaccine through school health services, with catch-up 3-dose vaccination offered to girls ages 13-18.

^4^Belgium: HPV vaccines started to be reimbursed in 2007 for certain cohorts in some regions by some sick funds. They have been fully reimbursed since December 2008 for all women aged 12 to 18 years

^5^Denmark: The 4vHPV vaccine was licensed in October 2006 and introduced into the children’s vaccination program in January 2009 for 12 year old girls, catch-up program for girls born in 1993–1995, starting in October 2008. From August 2012, another catch-up program targeted girls and women born in 1985–1992. Vaccine is administered free of charge by general practitioners to the birth cohorts covered by the programs.

^6^Ontario, Canada: Ontario began offering all 3 doses of the vaccine, free-of-charge, to all grade 8 girls in September of 2007. Doses are administered primarily through school-based immunization clinics, but girls also may receive the vaccine from a physician or at their health unit at no cost. Before September 2012, eligible girls had until the end of their grade 8 year to initiate the vaccine series and until the end of grade 9 to complete it. During the study period, girls who were not eligible for the school-based program (e.g., in grade 8 before 2007) could obtain the vaccine series for ∼$400.

^7^France: In 2007 in France, HPV vaccination was initially recommended for 14 year old females (primary cohort) and for those aged 15–23 years old (as catch-up) who had never had sexual intercourse or within the first year following sexual debut. In September 2012, French recommendations were updated and HPV vaccination is now recommended in 11 to 14 year old young women (primary cohort), with a catch-up for those aged 15–19 years.

^8^New Zealand: A government funded vaccination program against human papillomavirus (HPV) infection started in New Zealand in September 2008. After an initial catch-up phase, vaccination with a quadrivalent vaccine was routinely offered from year 2011 onwards to girls in school year 8 (typically age 12 years) or in primary care settings at age 12.

**Table S3. Variables Potentially Influencing Estimates of Impact and Effectiveness.**

| **Factors** | **Relevant Considerations** | **Examples of How Factor Might**  **Potentially Bias Estimates** |
| --- | --- | --- |
| Vaccination Program | - Percent uptake - Age at vaccination - Catch-up cohort - Length of time implemented | - Higher uptake –possibility of increased effectiveness/impact due to herd protection - Older age at vaccination- possibly decreased impact due to prior HPV exposure |
| Vaccination Status | - Data sources: registry, self-report, medical record review, insurance claims data - Identification of dose number - Identification of age at each dose | - Registry data can be more accurate than self-report - Dose number, date, and age at dose can help more accurately characterize timing of vaccination relative to sexual debut |
| Outcome Data | - Data sources: registry, self-report, medical record review, insurance claims data - Data type: (e.g., HPV infection – assay used; type-specific data availability; cervical screening outcome; histology data- pathology panel consensus diagnosis) | - Pap/Histology outcome categorization (CIN2, CIN2/3, CIN2+, CIN3, CIN3+) - HPV typing of cervical lesions can identify those attributed to 4vHPV vaccine types (HPV 16/18) |
| Other Factors | - Location of study - Secular changes in sexual behavior - Availability of risk factor data | - Secular trend such as decrease in average number of sex partners can independently be associated with lower rates of lesions (not necessarily attributable to vaccination) |
| Cervical Screening Program | - Secular changes in screening program - Population coverage - Age at screening start - Screening interval - Ability to link screening dates to vaccination dates | - Increase in screening over time can result in increased lesion detection and increased incidence due to secular trend rather than vaccination impact - Shorter screening interval result in increased incidence due to increased probability of detect lesion rather than vaccination impact |
| Observational Study Design | - Ecological, case-control, cohort - Impact/effectiveness | - Person-level data with vaccination and outcome status can help confirm/inform ecological data |
| Cohort Selected | - Screened/total population - Population-based - High risk population - Convenience sample - Comparison cohort- similar to vaccinated cohort - Potential biases among cohorts with incomplete vaccination series | - Total population includes those not screened - outcomes cannot be measured unless screened - Effectiveness measures can be influenced if individuals with incomplete vaccination series are at higher risk of HPV exposure prior to vaccination |
| Statistical Analysis | - Method - Sample size - Length of follow-up - Loss to follow-up - Availability of adjustment factors | - Smaller sample size - less stable estimates or reduced ability to identify actual impact - Method- raw or adjusted prevalence ratios; raw or adjusted relative risks using vaccination status data |

**Table S4. Summary of Impact on Genital Warts: % Reduction in Prevalence in Vaccine Era vs Pre-vaccine Era, by Country.**

| **Country** | **Reference** | **Setting** | **Data collection years, (number of years after vaccination program introduced) ^a^** | **Age-group**  **in women targeted by vaccination program** | **% GW reduction vs pre-vaccine era (95% CI)**  **In women targeted by vaccination program** | **Periodicity of reduction after vaccine introduction ^b^** | **Estimated Vaccine uptake** | **Herd-protection in men, statistically significant**  **Y/N/NA** |
| --- | --- | --- | --- | --- | --- | --- | --- | --- |
| Australia | Fairley 2009 [A29] | Melbourne Sexual Health Centre, Victoria, Australia | 2004-2008 (1) | <28 yo | 25.1% (19.3-30.5) | Per quarter | Approx. 70% for women <26 yo in 2008 | Y |
|  | Read 2011 [A37] |  | 2004-2011 (4) | <21 yo | 56%* *[OR = 0.44]* | Yearly |  | Y |
|  |  |  |  | 22-29 yo | 30%* *[OR=0.70]* | Yearly |  |  |
|  | Chow 2015 [A27] |  | 2004-2014 (7) | <21 yo | 45%* *[aOR**: 0.55]* | Yearly |  | Y |
|  |  |  |  | All | 22%* *[aOR** : 0.78]* | Yearly |  |  |
|  | Donovan 2011 [A28] | National surveillance network, patients attending sexual health services | 2004-2009 (2) | 12-26 yo | 59% (54–61) | Over study period (2 yrs post vaccination) | About 80% 12-17 yo; 64% 18-19 yo; 52% 20-26 yo (women) in 2010 | Y |
|  | Ali 2013 [A24] |  | 2004-2011 (4) | <21 yo | 92.6% | Over study period (4 yrs post-vaccination) |  | Y |
|  |  |  |  | 21-30 yo | 72.6% |  |  |  |
|  | Ali 2013 [A23] | In-patient treatment for genital warts in all private hospitals of Australia (Medicare) - nationwide | 2000-2011 (4) | 15-24 yo | 85.3% | Over study period (4 yrs post-vaccination) |  | Y |
|  | Harrison 2014 [A31] | Nationwide health facility activity registers of general practice | 2002-2012 (5) | 15-27 yo | 61% | Over study period (5 yrs post-vaccination) |  | N |
|  | Liu 2014 [A34] | Nationwide phone survey based on self-declaration | 2001-2011 (4) | 18-30 yo | 41% | Over study period (4 yrs post-vaccination) |  | NA |
|  | Smith 2014 [A39] | Hospital admissions for GW- from National Hospital Morbidity Database (public + private hospitals) | 1999-2011 (4) | 12-17 yo | 89.9% (84.62-93.4) | Over study period (4 yrs post-vaccination) |  | Y |
|  |  |  |  | 18-26 yo | 72.7% (67.0–77.5) |  |  |  |
|  |  |  |  | 27-30 yo | 42.1% (26.1–54.6) |  |  |  |
|  | Smith 2016 [A43] |  | 2004-2011 (4) | 10-19 yo | 85%-86.7% | Over study period (4 yrs post-vaccination) |  | Y |
|  |  |  |  | 20-29 yo | 61.6%-66% |  |  |  |
| Denmark | Baandrup 2013 [A25] | Hospitalization and outpatient visits for GW as assessed from the Danish National Patient Register | 1995-2011 (3) | 12-15yo | 51.9% (20.4-74.6) | Yearly | Between 80-85%, women <18 yo in 2011  By July 2013, 87-91% among 13-17 yo women In January 2014, 75% in women 27-29 yo | N |
|  |  |  |  | 16-17 yo | 45.3% (33.3- 55.8) |  |  |  |
|  |  |  |  | 18-19 yo | 14.4% (3.6- 24.4) |  |  |  |
|  | Sando 2014 [A38] | GW treatment prescription from Register of Medical Products Statistics combined  with data inpatient/  outpatient data from the National Patient Register | 2001-2011 (3) | 15-19 yo | 67% (63–72) | Over study period (3 yrs post-vaccination) |  | Y |
|  | Bollerup 2016 [A42] |  | 2006-2013 (5) | 12-15 yo | 42.7% (33.7-50.5) | Yearly |  | Y |
|  |  |  |  | 16-17 yo | 55.1% (51.2-58.7) |  |  |  |
|  |  |  |  | 18-19 yo | 39% (33.5-44) |  |  |  |
|  |  |  |  | 20-21 yo | 20.9% (16.3-25.2) |  |  |  |
|  |  |  |  | 22-25 yo | 11.7% (8.5-14.8) |  |  |  |
|  |  |  |  | 26-29 yo | 5.5% (3.9-7.2) |  |  |  |
| France | Judlin 2015 [A32] | Prospective multicenter study from gynecologic practices | 2008-2012 (5) | 15-18 yo | 47.1%*, SS  *[decrease from 0,34% to 0,18%]* | Over study period (4-5yrs post-vaccination) | Approx. 30%, women 15-17 yo in 2011 | NA |
| USA | Bauer 2012 [A26] | Claims data from the California  Family Planning Access Care and Treatment  Program (visit or pharmacy dispensing) | 2007-2010 (3) | <21 yo | 34.8% (31.5-38.2 ) | Over study period (3 yrs post-vaccination) | 49%, 13-15 yo women in 2010; 21% 19-26 yo women in 2010 | Y |
|  |  |  |  | 21-25 yo | 10.0% (6.3-13.6) |  |  |  |
|  | Flagg 2013 [A30] | Claims data (inpatient/  outpatient visits or pharmacy dispensing) from large claims database (Truven Health Analytics) | 2003-2010 (3) | 10-14 yo | No change | Over study period (3 yrs post-vaccination) | 43.5-54.6%, 13-17 yo women in 2010 | Y/N** |
|  |  |  |  | 15-19 yo | 38%*, SS [decrease from 2.9 to 1.8 per 1 000 pyrs] |  |  |  |
|  |  |  |  | 20-24 yo | 13%*, SS [decrease from 5.5 to 4.8 per 1 000 pyrs] |  |  |  |
|  | Nsouli-Maktabi, 2013 [A41] | Healthcare encounter with GW diagnosis within the Defense Medical Surveillance System | 2000-2012 (5) | <21 yo | 40%* *[From 3575.6 in 2006 to 2143.2 cases per 100 000 pyrs in 2012]* | Over study period (5 yrs post-vaccination) | Not stated | Y/N** |
|  |  |  |  | 21-24 yo | 25%* *[From 2700.2 in 2006 to 2017.3 cases per 100 000 pyrs in 2012]* |  |  |  |
| Canada | Smith 2015, Pediatrics [A20] | Ontario's administrative health database (inpatient/  outpatient visits) | 2005-2009 (2) | 14-17 yo | 19%*, NSS  [RR=0.81, 0.52  to 1.25] | Over study period (2 yrs post-vaccination) | 50.6% in study population | NA |
| Belgium | Dominiak-Felden 2015 [A17] | Large Belgian reimbursement database (MLOZ sick fund) | 2006-2013 (6) | 16-22 yo | 72.1% (64.7; 77.9 ) | Over study period (6 yrs post-vaccination) | 48% | Y |
| Sweden | Leval 2012 [A33] | Prescribed drug register and National Patient register in Sweden | 2006-2010 (3) | 17-18 yo | >25%, SS | Over study period | 25% 13-20 yo women; 30% in 15-18 yo women in 2011 | N |
|  |  |  |  | 15-25 yo | SS decreasing trend |  |  |  |
| Germany | Mikolajczyk, 2013 [A35] | German pharmaco-epidemiological research database. Data from a large health insurance company (inpatient, outpatient, and prescriptions) | 2005-2008 (1) | 15-19 yo | 23%, SS | Over study period (1 yr post-vaccination) | 40%, 16-18 yo women in 2008/  2009 | N |
|  |  |  |  | 16 yo | 47%, SS |  |  |  |
|  |  |  |  | 17 yo | 45%, SS |  |  |  |
|  |  |  |  | 18 yo | 35%, SS |  |  |  |
| New Zealand | Oliphant 2011 [A36] | Patient attending Auckland Sexual Health Services | 2007-2010 (2) | <20 yo | 62.8%*, SS  [from 13.7% in 2007 to 5.1% in 2010] | Over study period  (2 yrs post-vaccination) | School program: 51.7% in 2009 | Y |
|  | Wilson 2014 [A40] | National prescription data from the government's pharmaceutical purchasing agency | 1998-2012 (4) | <20 yo | 24.5%*, SS *[from 15.1% in 2007/2008 to 11.4% in 2011/2012]* | Over study period (4 yrs post-vaccination) |  | Not specified |
| a: For each study reported in this table, a consistent approach was undertaken to estimate the “number of years after vaccine program introduced”. The number of vaccine era data collection calendar years reported in each study was summed, and the year in which the vaccine program was first implemented was excluded.  b: expresses the periodicity of the % reduction in genital warts mentioned in the previous column (e.g., In Fairley 2009, a reduction of 25.1% per quarter was observed in the post-vaccination period).  *: recalculated from figures in paper [original figures]; **: adjusted for number of sexual partners; *** inconsistent decrease over study period, meaning that a statistically significant decrease was observed during some study periods but not during whole study period.  SS : statistically significant; NSS : non-statistically significant; Y: yes, N: no, NA: not applicable (since the study was conducted in females exclusively). | | | | | | | | |

**Table S5. Summary of Vaccine Effectiveness (VE) against Genital Warts (by dosing schedule and age group).**

|  | **Country** | **Reference** | **Estimate of vaccine effectiveness (95% CI)** | **Age group** |
| --- | --- | --- | --- | --- |
| **Population targeted by vaccination program/youngest cohorts**  **(VE for full 3 doses schedule)** | Denmark | Blomberg 2013 [A21] | 88%* (64;96) | 1995-96 cohort |
|  | Sweden | Herjweier 2014 [A18] | 82%*(78;85) | 10-16 years old |
|  |  | Leval 2013^1^ [A19] | 76% (73;79) | 20-44 years old, first dose before 20 years old |
|  |  |  | 93% (73;98) | Youngest cohort, (vaccination before 14 years old) |
|  | Canada | Smith 2015^2^ [A20] | 44% (35;76)^a^ – 66%* (-114 ;95)^b^ | 14-17 years old |
|  | Belgium | Dominiak-Felden 2015 [A17] | 88% (79.4; 93.0) | 16-22 years old |
| **Influence of age/age at vaccination** | Denmark | Blomberg 2013 [A31] | 88%* (64;96) | 1995-96 cohort |
|  |  |  | 78%*(67;85) | 1993-1994  cohort |
|  |  |  | 75%* (68;81) | 1991-1992 cohort |
|  |  |  | 38%* (24;50) | 1989-90 cohort |
|  | Sweden | Leval 2013 [A19] | 93% (73;98) | <14 years old at vaccination |
|  |  |  | 80% (75;83) | 14-16 years old at vaccination |
|  |  |  | 71% (65;76) | 17-19 years old at vaccination |
|  |  |  | 48% (22;65) | 20-22 years old at vaccination |
|  |  |  | 21%, NSS | 23-26 years old at vaccination |
|  | Belgium | Dominiak-Felden 2015 [A17] | 89.0 (73.2; 95.5) | <15 years old at vaccination |
|  |  |  | 90.4 (78.3; 95.7) | 15-17 years old at vaccination |
|  |  |  | 68.5% (1.2; 89.9) | ≥18 years old at vaccination |
|  | US | Swedish 2012** [A22] | 55% (0.8;78) | 42 years old (mean age) |
| **Influence of schedule completion**  **(3 doses as full schedule)** | Sweden | Herjweijer 2014 [A18] | 82%*(78;85) - 3 vs 0 dose  71%*(60;79) - 2 vs 0 dose  69%*(51;80) - 1 vs 0 dose | 10-16 years old |
|  | Belgium | Dominiak-Felden 2015 [A17] | 88.0% (79.4; 93.0)- 3 vs 0 dose  65.7% (16.9; 85.9)- 2 vs 0 dose  36.6% (-16.1; 65.4)- 1 vs 0 dose | 16-22 years old |
|  | Denmark | Blomberg 2015 [A16] | 49%*, SS - 1 vs 0 dose  56%*, SS - 2 vs 1 dose  54%*, SS - 3 vs 2 doses*** | Cohorts 1985-1999 |
| *VE recalculated from original figures as : VE = 1-IRR, VE=1-RR, VE=1-HR or VE=1-OR  **Study conducted in men who have sex with men.  ***When time interval between the 2 doses was >6 months, IRR for 3 vs 2 doses approximated 1.  1: In this study, the actual age-group of females considered in VE analysis was not stated; therefore we chose to present both the overall main VE results, corresponding to women who had been vaccinated before 20 years old and the VE estimate in the youngest cohorts, corresponding to girls vaccinated before 14 years old.  2: The methodological approach used in that study was different compared to the other studies (regression discontinuity).  a: evaluation based on nonspecific case definition for genital warts (*i.e*., diagnostic code used to identify genital warts could also have been used to capture warts on other body sites) as in Table 3 of publication.  b: evaluation based on more specific case definition for genital warts (*i.e*., evaluated as "probable" case according to authors) as in Table 3 of publication. | | | | |

**DETAILED FIGURE LEGENDS for Figures 2 and 3.**

**Figure 2. Impact and effectiveness of 4vHPV vaccination on prevalence of vaccine genotypes.**

***Australia*** (Chow 2015 [A27], impact): Prevaccine era (2004-2007, n=160 Australian-born); first 7 years of vaccine era (2007-2014, n=468 Australian born, 80% received ≥1 vaccine dose, proportion vaccinated prior to start of sexual activity not reported but population ~70% coverage in females ≤21 years old in 2013-2014 or 14 years old in 2007-2008 likely prior to start of sexual activity), high-risk population (females diagnosed with *Chlamydia trachomatis* infection from Melbourne Sexual Health Centre), ≤25 years old (median age 22 years; high proportion of females <22 years would likely have been vaccinated), self-reported vaccination status, vaccinated implies ≥1 dose; clinician-collected specimens; prevalence ratios adjusted for potential confounders.

***Australia*** (Tabrizi 2014 [A5], impact & effectiveness): Prevaccine era (2005-2007, n=202); first 6 years of vaccine era (2010-2012, n=1058, 86% ≥1 dose; 48% of vaccinated population reported sexual activity prior to vaccine series completion); representative sample of females receiving Pap screening at family planning clinics in Sydney, Melbourne, and Perth; 18-24 years old, mean age 21 years; self-reported vaccination status verified against National Vaccine Program Register; clinician-collected specimens; results from the vaccine era are based on small numbers of cases positive for HPV 6 (not shown) or HPV 11 (3 positive samples in prevaccine era; 4 positive samples postvaccination era); prevalence ratios and vaccine effectiveness adjusted for potential confounding variables; vaccinated defined as ≥1 dose unless otherwise noted.

***Belgium*** (Merckx 2014 [A14], impact): Vaccine era only (2009-2012, n=3395 <19 years old; n=22,137 females, 19-24 years old); since 2007 vaccine reimbursed for 12-15 year old females and extended to 18 year olds; cervical screening population; no vaccination status information; clinician-collected specimen; sample 15-24 years old by 2012; not shown: HPV 18, 20-24 year olds, RR: 1.23, 95% CI (0.89-1.69); 85% of females vaccinated in Flemish region (~90% of those vaccinated likely had 4vHPV vaccine); no adjustment for potential confounding variables, unknown proportion vaccinated prior to start of sexual activity; Poisson regression trend analysis.

***Germany*** (Delere 2014 [A2], effectiveness): Vaccine era [2010-2012, n=223 vaccinated (87% 4vHPV vaccine, 89% had 3 doses; only 31% reported no sexual activity prior to vaccination), n=512 not vaccinated]; population-based cross sectional study (12% response rate- possible self-selection bias); 48% had at least one cervical screening; 20-25 years old, mean age 23 years; self-reported vaccination status; self-sampled specimens; vaccinated defined as ≥1 dose (89% of whom had 3 doses). Unadjusted PR shown in table; not shown is a separate multivariate adjusted analysis showing 56% reduction in 16/18 infections in 20-21 year olds.

***Sweden*** (Soderlund-Strand 2014 [A13], impact): Pre-vaccine program sample (2008; n=44,146); ~4-5 years of vaccine era (2013, n=5815) (vaccine was available starting in 2006 & was subsidized for on-demand vaccination for females 13-17 years old; organized publicly-funded program for 10-18 year olds launched in 2012); large population-based study of 13-22 year old females (most 18-22 years old) undergoing routine *Chlamydia trachomatis* screening; in study sample, vaccine coverage for 18-22 year olds in pre-vaccine program era ranged from 2%-17% (resultant misclassification would bias results toward null), and in vaccine era coverage was 53% (females 13-20 years old), 35% (21 years old), and 29% (22 years old); vaccination status from registry; clinician-collected specimen; unadjusted estimates; no individual sexual behavior information.

***US*** (Cummings 2012 [A1], effectiveness): Prevaccine era (1999-2005, n=150), vaccine era (2010, n=75, 89% ≥1 dose, 78% ≥2 doses; 27% of females receiving ≥2 doses reported sexual activity prior to vaccination); small sample of high-risk urban minority population attending primary care clinic; 75% sexually active; ~90% African American; 14-17 years old; mean age at first sexual activity 14 years; clinician or self-sampled specimen – possible bias); apparently clinician-verified vaccination status; for analysis of HPV 16/18, prevaccine era prevalence compared to vaccinated (≥1 dose); odds ratios from logistic regression incorporating random pair effects.

***US*** (Dunne 2015 [A10], impact): Prevaccine era (2007, n=4138), first 6 years of vaccine era (2012/2013, n=4171, 32% ≥1 dose, ~25% 3 doses, 47% age ≥19 years at first dose; proportion vaccinated prior to start of sexual activity not reported); screened and insured population; 71% white 20-29 years old (~50% <25 years old); not shown in figure: females whose age at first dose <19 years associated with decreased 4vHPV type prevalence by 90% compared to unvaccinated; vaccination status from electronic medical records; clinician-collected cervical specimen; figure shows unadjusted prevalence ratios.

***US*** (Kahn 2012 [A11], impact): Prevaccine era (2006-2007, n=368), vaccine era (2009-2010, n=409, 59% ≥1 dose; proportion vaccinated prior to start of sexual activity not reported); high risk population, sexually active, 40% history of *Chlamydia trachomatis* infection, recruited from 2 primary care clinics; ~70% African American; age at first sexual intercourse ~15 years; vaccination status from State Immunization Registry for 87% of sample, 13% self-reported; clinician or self-sampled specimen- possible bias; mean age 19 years; propensity score adjusted prevalence.

***US*** (Markowitz 2016 [A7], impact and effectiveness): Prevaccine era (2003-2006, n=1363) first 6 years of vaccine era (2009-2012, n=736 for ages 14-19 years, n=470 for ages 20-24 years); nationally representative samples from NHANES; 51% of 14-19 year old sample ≥1 dose, 33% of 20-24 year old sample ≥1 dose; results for HPV 11 in 20-24 year old females not shown- prevaccine era n=1; vaccine era n=0; results for combined 14-24 year old category limited to sexually active females: prevaccine era n=1092, vaccine era n=753, 38% ≥1 dose, vaccinated defined as ≥1 dose; self-reported vaccination status and self-collected specimen; adjusted prevalence ratios.

***US*** (Schlecht 2012 [A3], effectiveness): ~4-5 years of vaccine era (calendar years not specified); vaccinated (n=327 with 3 doses; n=186 with 1-2 doses; 90% reported sexual intercourse prior to 1^st^ dose) vs unvaccinated (n=132); sexually active high-risk minority population attending largest adolescent health clinic New York City; 92 % non-Caucasian ; 14-20 years old, mean age 18 years; 67% age at first intercourse <16 years; clinician-collected vaccination status (unclear if self-reported or from medical record); clinician-collected specimen; vaccinated defined as 3 doses; results derived from general estimating equation with multivariate logistic regression.

**Figure 3. Impact and effectiveness of 4vHPV vaccination on cervical cytological and histological abnormalities**

**Panel A. Australia: population-based analysis of percent reduction in cervical abnormalities among vaccinated (at least 1 dose) compared to contemporaneous unvaccinated screened females in Victoria.**

Studies included in analysis (N = 57)

HPV (n = 14)

Cervical lesions (n = 16)*

Genital warts (n = 28)*

*One study reported both outcomes

**CIN1 (Gertig 2013 [A46], effectiveness):** Population-based analysis of first 5 years of screening data following start of Australia’s HPV vaccination program; females aged 12-17 in 2007 (i.e., eligible for vaccination); vaccinated females (n= 24,871) had 194 CIN1 cases among 21,199 fully vaccinated females (3 doses); unvaccinated females (n= 15,192) had 244 CIN1 cases; analysis included females eligible for 2-year catch-up (aged 14-17); study time period: April 2007-December 2011; follow-up time: average 1.5 years, maximum: 4.8 years; mean age at vaccination 15.7 years; % reduction based on adjusted hazard ratios; some analyses had small sample sizes; screened at age 12-17 years suggests higher-risk population so may not be generalizable to overall population; country-level vaccine uptake as reported/referenced in publication: school-based vaccine uptake 86/82/75% for doses 1/2/3; results for at least 1 dose similar to results for 3 doses; vaccination and case status determined from registry data.

**All other data (Brotherton 2015 [A44]):** Population-based analysis of first 5 years of screening data following start of Australia’s HPV vaccination program; females aged <=26 in 2007 (i.e., eligible for vaccination) n=289,904; mean age in 2007 (at vaccine eligibility) was 21-22 yrs; 54% vaccinated with at least 1 dose & 37% with 3 doses; final vaccine dose prior to first cervical screening; follow-up: average 2.9 years, maximum: 4.8 years; study time period: April 2007-December 2011; % reduction based on adjusted hazard ratios (aHR) of vaccinated vs unvaccinated in vaccine era; results for at least 1 dose similar to results for 3 doses; screened at age <20 years suggests higher risk population so may not be generalizable to overall population; vaccination and case status determined from registry data.

**Panel B. Australia: population-based analysis of percent reduction in cervical abnormalities among vaccinated compared to contemporaneous unvaccinated screened females in Queensland.**

**Crowe 2014 [A45], effectiveness:** Population-based analysis of first 4 years of screening data following start of Australia’s 4vHPV vaccination program; case-control study nested in screening cohort; females aged 11-27 in 2007 (i.e., eligible for vaccination); first cervical screening outcome defined case/control status; controls (n=96,404) had normal cytology; “low grade/other” cases (n=10,887) had cytological abnormalities not confirmed by histology, low grade confirmed by histology, or high grade cytological abnormality without subsequent histological testing; “CIN2+/AIS” cases (n=1062) had CIN2, AIS, or worse; follow-up time: low-grade/other cases: 1.8 years, high-grade cases: 2.1 years, controls: 2.2 years; % reduction based on adjusted odds ratios (aORs); ages without bars have ≤5 cases, except for adjusted ages 23-27 years high grade cases with 1 dose: aOR 1.22 (0.89-1.7); small sample (n=6) of high grade cases with 3 doses among 11-14 year olds; school-based program uptake (aged 12-17): 84% (1 dose); 70% (3 doses); community program uptake (aged 18-26): 64% (1 dose); 33% (3 doses); vaccination and case status obtained from linked administrative health datasets; misclassification of dose number may have occurred- vaccination database inaccuracies noted in 12% of 12-18 year olds and 25% of 19-26 year olds.

**Panel C. Canada: percent reduction in cervical abnormalities in vaccinated/vaccine era compared to contemporaneous unvaccinated/prevaccine era in 3 provinces.**

**ASCUS+ and LSIL+ (Mahmud 2014 [A47], effectiveness), Manitoba:** Analysis of first 4 years of vaccine availability in screened population of females 15-17 years old and ≥18 years old at enrollment (2006-2010); vast majority would have been vaccinated outside the school-based vaccination program, which started in September 2008 targeting 11-12 year old females; unvaccinated n=9594; vaccinated= 3541; without prior abnormal Pap screening; median length of follow-up 3.1 years; vaccinated (any dose) versus unvaccinated in vaccine era; adjusted vaccine effectiveness estimates as reported in publication; ASCUS+: ASCUS or worse, LSIL+: LSIL or worse; vaccine uptake not reported; vaccination status and cytology outcomes identified from population-based registries/databases.

**Dysplasia (Smith 2015 [A20], impact and effectiveness), Ontario:**  Majority of mild dysplasia cases likely ASCUS, as reported in publication due to young age; n= 260,493 females; n=2436 incident cases; prevaccine era: 8^th^ grade in 2005/6 and 2006/7 school years; vaccine era: grade 8 in 2007/8 and 2008/9 school years; mean length of follow-up 4.6 years (*i.e*., follow up between grades 10 and 12); prevaccine era versus vaccine era results: 24% reduction for probable cases; 21% for broader case definition, irrespective of vaccination status); prevaccine era versus fully vaccinated (3 doses) results: 49% reduction for probable cases; 44% for broader case definition); vaccine-era females were age-eligible for vaccination; ~50% vaccine uptake in population; % reduction based on risk ratio from log-binomial regression; vaccination status and cytology outcomes identified from population-based registries/databases; cytology status imprecise; “program impact” defined as prevaccine versus vaccine era; “vaccine impact” defined as fully vaccinated (3 doses) versus prevaccine era.

**CIN2+ (Ogilvie 2015 [A58], impact), British Columbia**: Prevaccine era compared to up to 3 years after vaccine program implementation among females 15-17 years old (ages at Pap testing leading to diagnosis; group was age-eligible for vaccination) in vaccine era (small n=10 cases of CIN2+ among vaccine-eligible females 15-17 years old) versus pre-vaccine era; not shown: ages 18-22 years (not age-eligible for vaccination) had 1%-7% reduction in “CIN2+” (CIN2 and CIN3); vaccine uptake reported in publication was ~60% for 3 doses; lesion data from population-based cancer registry; % decrease based on “overall <18 years adjusted for age” rate ratios from piece-wise Poisson regression reported in Table 4 of publication; authors also report 90% reduction in this age group (unclear how these estimates differ); Ecological analysis – many potential confounding factors uncontrolled and individual-level vaccination status unknown; pap screening guidelines changed in 2011, resulting in 30-50% reduction in cytology specimens. This timing also corresponding with “vaccine era”, overlapping with years that birth cohorts become eligible for vaccination (2010-2012). During the vaccine era, a reduced number of cervical specimens were collected in comparison to the prevaccine era, possibly reflecting a population at higher risk of CIN2+ in the vaccine era.

**Panel D. Denmark: percent reduction in cervical abnormalities in females vaccinated with 4vHPV vaccine (≥1 dose) versus unvaccinated women by birth cohort.**

**Baldur-Felskov, 2014 [A48], effectiveness**: Study period: 2006-2012; nationwide study; n=399,244 females, 247,313 of whom were vaccinated; small n for CIN3+ 1993-94 cohort; birth cohorts 1993-94 and 1995-96 (eligible for childhood & catchup programs; birth cohorts 1989-90, 1991-92 (vaccination not covered by national program), vaccinated (≥1 dose) vs contemporaneous unvaccinated; n=3629 atypia+ (atypical cytology or worse, without a histological endpoint); n=708 CIN2+ (CIN2, CIN3, CIS, AIS); n=365 CIN3+= CIN3, CIS, AIS. % reduction based on unadjusted hazard ratios (no control for potential confounding factors); uptake as noted in publication: 1989-1990 birth cohort was 14%; 1991-1992 birth cohort was 27%; 1993-1999 birth cohort was >85%; results shown for total population (screened and unscreened); small number of screened females (126) in 1995-96 cohort); vaccination status and lesion diagnoses determined from national registers.

**Panel E. Sweden: percent reduction in CIN2+ and CIN3+ among females fully vaccinated with 4vHPV vaccine (3 doses) versus unvaccinated/partially vaccinated females, by age at first dose.**

**Herweijer 2016 [A50], effectiveness:** Study period: 2006-2013; nationwide study; all resident females 13-29 years old at vaccination initiation (n=1,333,691 females; 18% were vaccinated of which 77% had 1^st^ dose before age 17; Total (vaccinated & unvaccinated females) CIN2+ n=22,616 & CIN3+ n=12,645; cases among vaccinated females shown in figure; small n for CIN3+ <17 years old); followed up to 8 years after 4vHPV vaccine introduction; mean follow-up in unvaccinated (5.1 years) and vaccinated (2.6 years); CIN2+ = CIN2, AIS or worse; CIN3+ = CIN3, AIS or worse; % reduction based on adjusted incidence rate ratios from Poisson regression, incorporating 6-month lag time (*i.e*., females vaccinated within 6 months of diagnosis were considered “unvaccinated” in the analysis); results include screened and unscreened females; routine screening age is 23 or older (bars for <17 and 17-19 year olds at diagnosis are mostly pre-screening age population; bars for 20-29 year olds are mostly screening population); vaccine uptake noted in publication as of December 2014 was 82%, 1 dose (10-12 year olds) and 60% (13-18 year olds); comparison group is partially vaccinated and unvaccinated; vaccination status and lesion diagnoses determined from national registers.

**Panel F. US: percent reduction in HPV 16/18-related cervical abnormalities among females vaccinated with 4vHPV vaccine (at least 1 dose) compared to contemporaneous unvaccinated females, by time between first dose and screening test leading to diagnosis.**

**Hariri 2015 [A49, A55], effectiveness:** Analysis of first 5 years of screening data (2008-2012) following 4vHPV vaccine availability using data from US CDC’s HPV-IMPACT Project(population-based surveillance in 5 catchment areas in US); CIN2+ n=549 vaccinated (≥1 dose before >12 months prior to pap test) 152 of whom had CIN3/AIS, 1274 not vaccinated 427 of whom had CIN3/AIS; among females ≤26 years old in 2006 (*i.e.,* eligible for vaccination); median age at CIN diagnosis: 23 years among vaccinated & 25 years among unvaccinated; “CIN2+” = CIN2, CIN2/3, CIN3, AIS; “CIN3/AIS” is subset of CIN2+; % reduction based on adjusted prevalence ratios (aPR), comparing vaccinated (any dose) to unvaccinated using log binomial regression, by time between vaccination and pap test resulting in CIN diagnosis (longer intervals assumed to be indirect measure of vaccination occurring prior to infection with HPV type responsible for CIN); vaccination status obtained from multiple sources; 2215 lesions not included in analysis because vaccination status unknown; medical record confirmed CIN diagnoses, HPV typed & only included 16/18-related lesions; not shown: aPRs for interval 1-12 months CIN2+ and CIN3/AIS were 1.02 (0.87-1.19) and 1.17 (0.97-1.40), respectively, and for 25-36 months CIN3/AIS: 1.02 (0.83-1.25).
